# FGL2-targeting T cells exhibit antitumor effects on glioblastoma and recruit tumor-specific brain-resident memory T cells

**DOI:** 10.1038/s41467-023-36430-2

**Published:** 2023-02-10

**Authors:** Qingnan Zhao, Jiemiao Hu, Lingyuan Kong, Shan Jiang, Xiangjun Tian, Jing Wang, Rintaro Hashizume, Zhiliang Jia, Natalie Wall Fowlkes, Jun Yan, Xueqing Xia, Sofia F. Yi, Long Hoang Dao, David Masopust, Amy B. Heimberger, Shulin Li

**Affiliations:** 1 Department of Clinical Pharmacy, Shanghai General Hospital, Shanghai Jiao Tong University School of Medicine, Shanghai, 200020 China; 2 Shanghai Key Laboratory of Pancreatic Disease, Shanghai Jiao Tong University School of Medicine, Shanghai, 201620 China; 3 Division of Pediatrics, The University of Texas MD Anderson Cancer Center, Houston, TX 77030 USA; 4 Uaub Institute for Research on Alzheimer’s Disease and the Aging Brain, Columbia University Irving Medical Center, New York, NY 77030 USA; 5 Department of Bioinformatics and Computational Biology, The University of Texas MD Anderson Cancer Center, Houston, TX 77030 USA; 6 Department of Neurological Surgery, Malnati Brain Tumor Institute of the Lurie Comprehensive Cancer Center, Feinberg School of Medicine, Northwestern University, Chicago, IL 60611 USA; 7 Department of Veterinary Medicine & Surgery, The University of Texas MD Anderson Cancer Center, Houston, TX 77030 USA; 8 Beijing Institute of Brain Disorders, Capital Medical University, Beijing, 100069 China; 9 Department of Microbiology, Center for Immunology, University of Minnesota, Minneapolis, MN 55455 USA

**Keywords:** Cancer immunotherapy, CNS cancer, T cells, Tumour immunology

## Abstract

Although tissue-resident memory T (T_RM_) cells specific for previously encountered pathogens have been characterized, the induction and recruitment of brain T_RM_ cells following immune therapy has not been observed in the context of glioblastoma. Here, we show that T cells expressing fibrinogen-like 2 (FGL2)–specific single-chain variable fragments (T-αFGL2) can induce tumor-specific CD8^+^ T_RM_ cells that prevent glioblastoma recurrence. These CD8^+^ T_RM_ cells display a highly expanded T cell receptor repertoire distinct from that found in peripheral tissue. When adoptively transferred to the brains of either immunocompetent or T cell-deficient naïve mice, these CD8^+^ T_RM_ cells reject glioma cells. Mechanistically, T-αFGL2 cell treatment increased the number of CD69^+^CD8^+^ brain-resident memory T cells in tumor-bearing mice via a CXCL9/10 and CXCR3 chemokine axis. These findings suggest that tumor-specific brain-resident CD8^+^ T_RM_ cells may have promising implications for the prevention of brain tumor recurrence.

## Abstract (web summary)

Glioblastoma is an aggressive type of cancer with poor patient prognosis. Here, the authors show that T cells armed with an FGL2-specific scFV can induce antitumour responses mediated by tissue-resident memory T cells in the brain.

## Introduction

Memory T cells provide rapid and effective immune protection against a wide variety of antigens, including pathogens and malignant tumors. Memory T cells consist of two major populations: non-recirculating resident memory T (T_RM_) cells^1–3^ and recirculating memory T cells^4^. Recirculating memory T cells include effector memory T cells, central memory T cells, and migratory memory T cells. Research has shown that T_RM_ cells are more potent effectors than recirculating memory T cells^5–8^. T_RM_ cells, often bearing CD69 and the αEβ7 integrin (CD103), and lacking CD62L expression, provide superior immunity to localized infection^9^. CD103 binds to epithelial E-cadherin, while CD69 blocks T cell egress via inhibition of the function of sphingosine-1-phosphate receptor-1 (S1PR1)^10^. The expression of CD103 and CD69 mediates the retention of T_RM_ cells in tissue. CD62L is a lymphoid homing molecule that helps peripheral T cells home to secondary lymphoid organs. To date, T_RM_ cells have been found in both barrier and non-barrier tissue types, such as the skin^11,12^, brain^13^, lung^14–18^, liver^19^, and breast^3^, where they mediate long-lived protection against reinfection. However, whether induction of T_RM_ cell formation in tumor tissue, such as glioblastoma (GBM), can lead to tumor shrinkage and prevent recurrence has not been examined extensively.

As a member of the fibrinogen-like protein family, fibrinogen-like 2 (FGL2) possesses prothrombinase activity and immune regulatory functions in both viral infection and cancer development. Accumulating evidence shows that FGL2 acts as an immunosuppressive regulator of B cell, T cell, and dendritic cell (DC) functions by binding to FcγRIIB and regulating adaptive immunity via Th1- and Th2-type cytokines^20^. Our previously published data showed that overexpression of FGL2 correlates with upregulated expression of negative immune checkpoints, decreased granulocyte–macrophage colony-stimulating factor–induced CD103^+^ DC differentiation, faster glioma progression, and poor clinical outcomes in brain malignancies^20–23^.

An analysis of data from The Cancer Genome Atlas found an inverse correlation between FGL2 expression and GBM patient survival^22^. As such, FGL2 is an attractive target for brain tumor immunotherapy. Indeed, FGL2-specific polyclonal antibodies induce antitumor activity against GBM tumor cells in syngeneic mouse models. However, owing to their poor blood–brain barrier penetration, the potential of FGL2-blocking antibodies to suppress brain tumor progression is limited.

Here, to improve the efficacy of FGL2 blockade for glioma treatment, we generate T cells armed with an FGL2-blocking single-chain variable fragment (scFv). Compared with control T cells (T-Ctr), T cells bearing this FGL2-blocking scFv (T-αFGL2) show superior antitumor effects without inducing obvious toxicity at the therapeutic dose. Importantly, the long-term mouse survivors in the T-αFGL2 treatment group generate tumor-specific brain-resident memory CD8^+^ T (CD8^+^ T_RM_) cells that reject rechallenge with tumor cells in the brain. These CD8^+^ T_RM_ cells are CD69^+^CD8^+^ T cells that display an expanded T cell receptor (TCR) repertoire. Of note, the CD8^+^ T_RM_ cells can be transplanted into the brains of naïve mice to convert these naïve mice into CD8^+^ T_RM_-bearing mice. The T-αFGL2 treatment boosts the CD69^+^CD62L^−^CD8^+^ T cell population, and this effect is abolished upon either depletion of the CXCR3 ligands CXCL9/10 or knockout of CXCR3 in the host mice, revealing an unanticipated link between CXCL9/10-CXCR3 signaling and tumor-specific CD8^+^ T_RM_ cell formation in brains.

## Results

### T-αFGL2 treatment has limited antitumor cytotoxic T lymphocyte activity in vitro

**Fig. 1:**
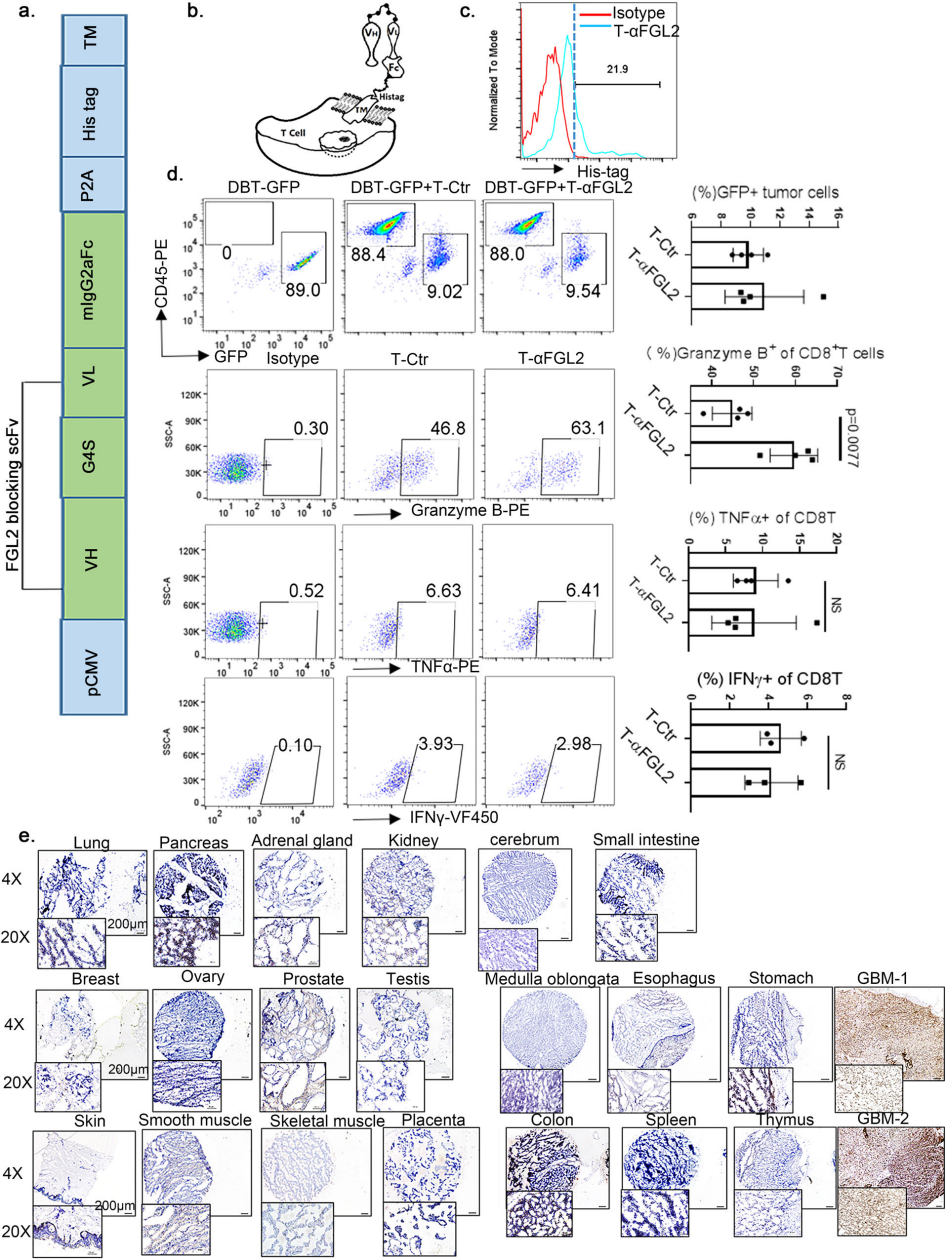
Restricted functional antitumor activity of T-αFGL2 cells in vitro. **a** Schematic of the vector encoding the FGL2-blocking scFv. **b** Schematic of a T cell transfected with the FGL2-blocking scFv vector (T-αFGL2). **c** Representative flow cytometry histograms demonstrating expression of the FGL2-blocking scFv on mouse T cells following transduction. **d** Flow cytometry plots depicting no difference in proportion of tumor cells (DBT-GFP+) cocultured with T-Ctr or T-αFGL2 cells at E:T ratio of 4:1 for 72 h (top panel); flow cytometry plots depicting higher granzyme B expression and no difference in TNFα or IFNγ expression in T-αFGL2 cells (compared with T-Ctr) cocultured with DBT tumor cells at E:T ratio of 1:1 for 24 h. Data are mean ± SD from three independent experiments. NS not significant, two-tailed *t*-test. **e** Representative micrographs of FGL2 expression in GBM and the indicated normal human tissue samples. The images of normal human tissue shown are representative results from two samples. Micrographs are representative of two sections per tissue sample. Scale bars, 100 μm.

Extensive prior efforts by our group to develop a therapeutic antibody targeting FGL2 failed to prevent recurrence—the primary cause of brain tumor–associated death (https://patentscope.wipo.int/search/en/detail.jsf?docId=WO2018204928). To obtain effective FGL2-specific monoclonal antibodies (mAbs), we obtained 75 clones from three independent hybridoma fusions. In a murine ELISA FGL2 binding assay (Supplementary Fig. 1a), 13 of 75 clones showed strong binding activity to murine FGL2 but not to the His-tag. Human FGL2 was then used to select mAbs that showed bi-species binding reactivity (Supplementary Fig. 1b). Mouse FGL2 binding clone #4 showed high binding affinity to human FGL2 (Supplementary Fig. 1a, b) and the most linear association between binding capacity and dilution (Supplementary Fig. 1c). Western blotting, immunofluorescence staining, and ELISA further validated the binding activity of FGL2 mAb-clone #4 to both mouse and human FGL2 (Supplementary Fig. 1d–f). Extensive evaluation of this mAb for treating glioma were investigated but only very modest in vivo therapeutic efficacy was achieved and no protective immunity against recurrence was found (filed patent WO2018204928). To utilize this mAb for blocking Fgl2, we proposed to use T cells as a delivery vehicle in this study, which may change the microenvironment where the FGL2-blocking T cells migrate and also remove the inhibitory effect on the armed T cells. In this regard, we further tested the effect of the FGL2-blocking scFv. Lentiviral constructs derived from FGL2 mAb-clone #4 scFv were generated to arm T cells (Fig. 1a). This construct contained scFv domains that recognize and block FGL2 (Fig. 1a). To ensure flexibility of the FGL2 scFv on the surface of T cells, an EGFR transmembrane domain was linked to the FGL2 scFv by a P2A linker (Fig. 1a, b). The expression of the FGL2 scFv on the T cell membrane was validated by staining of the His-tag domain (Fig. 1c). The transduction efficiency of the mouse T cells was consistent and in the range of 15–25% (Fig. 1c). To verify that T-αFGL2 cells can directly block FGL2, a microfluidics chip binding assay was established. As shown in Supplementary Fig. 1g, T-αFGL2 cells directly bind the FGL2 that is anchored on the chip via a biotin-streptavidin covalent bond. The antitumor cytotoxic T cell activity of T-αFGL2 cells against the FGL2-expressing murine glioma cell line, DBT, was evaluated by measuring the proportion of live glioma cells and granzyme B^+^, interferon γ (IFNγ)^+^, and tumor necrosis factor α (TNFα)^+^ T cells (Fig. 1d). T-αFGL2 cells expressed higher granzyme B levels than did T cells transfected with a control construct (T-Ctr) when co-cultured with DBT cells at an effector-to-target ratio of 1:1. The T-αFGL2 and T-Ctr cells had comparable levels of IFNγ and TNFα. However, no significant difference was found in the proportion of glioma cells in co-culture with T-αFGL2 and T-Ctr cells, suggesting that T-αFGL2 may have limited direct tumor cell killing effects in vitro.

### T-αFGL2 treatment does not cause toxicity in immunocompetent mice

To evaluate the suitability of FGL2 as a target for T cell therapy with low risk of off-tumor on-target toxicity, we assessed the expression of FGL2 in human GBM and normal tissue arrays using FGL2 mAb-clone #4, from which the αFGL2 construct was derived. As shown in Fig. 1e, FGL2 was highly expressed in human GBM tissue but not in healthy medulla oblongata tissue samples. In healthy tissue arrays (Fig. 1e), major organs such as the brain, lung, breast, spleen, and muscle were FGL2 negative, while moderate expression of FGL2 was observed in the stomach, colon, and pancreas (Fig. 1e). To assess the potential toxicity of T-αFGL2, we intravenously injected 5 million T-Ctr or T-αFGL2 cells into non-tumor-bearing 7-week-old immunocompetent Balb/c mice. Five days after T cell injection, we evaluated blood chemistry, organ toxicities, and immune cell populations in the spleen and bone marrow. As shown in Supplementary Fig. 2a, b, mice treated with T-αFGL2 exhibited no significant changes in immune cell counts in either the spleen or bone marrow. T-αFGL2 treatment caused no abnormalities in blood chemistry (Supplementary Fig. 2c), but mice treated with T-Ctr had significantly higher blood levels of albumin (*P* = 0.0264) and globulin (*P* = 0.0181) than did untreated mice (Supplementary Fig. 2c). A board-certified veterinary pathologist (N.W.F.) observed no evident abnormalities or aberrant T cell infiltration in tissue sections following T-αFGL2 cell infusion (Supplementary Fig. 3 and Supplementary Table 1). Taken together, these results show that T-αFGL2 therapy does not cause detectable organ toxicity in immunocompetent mice.

### T-αFGL2 therapy induces superior antitumor activity in vivo

**Fig. 2:**
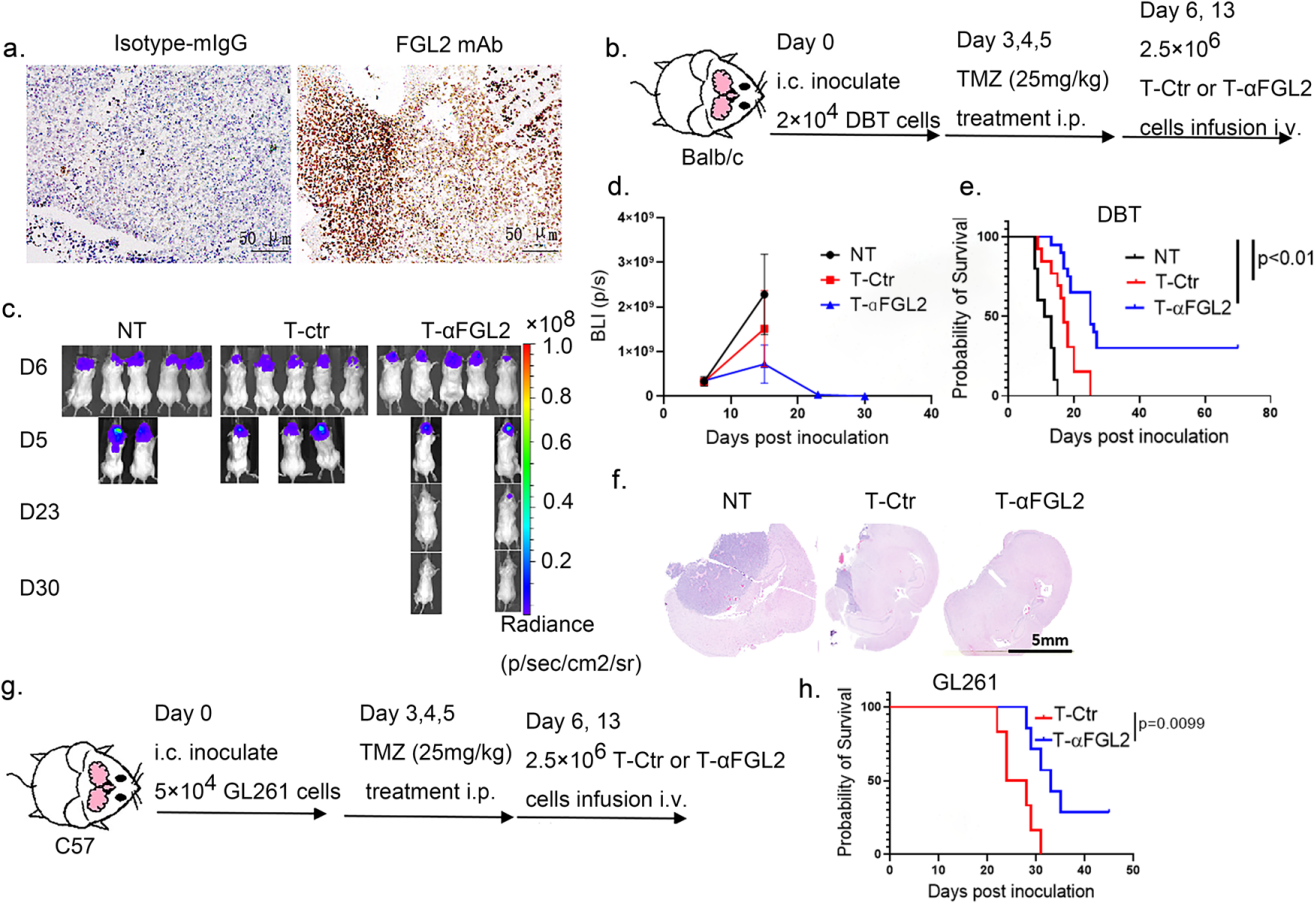
Antitumor activity of T-αFGL2 in vivo. **a** Representative micrographs of FGL2 expression in the brains of mice with glioma. Slides stained with mouse IgG were used as negative controls. Images are representative of results from three samples with multiple fields of view for each sample. **b** Schematic of the orthotopic DBT glioma model. On days 3–5 after DBT tumor cell inoculation, mice were treated with temozolomide (TMZ), followed by infusion of T-Ctr or T-αFGL2 cells on days 6 and 13. **c** Representative bioluminescence images of DBT-luc tumor growth in the orthotopic glioma model shown in (**b**). **d** Flux vs. time [p/s] data (mean ± SEM) for the orthotopic glioma model shown in (**b**) (*n* = 5 mice). **e** Kaplan–Meier survival curves of mice shown in (**b**) (*n* = 10 mice for NT group, *n* = 13 for T-Ctr group, *n* = 20 for T-αFGL2 group), log-rank test. The experiments were repeated three times with similar results. **f** Representative H&E staining of brains in (**b**) collected on day 14 after tumor cell inoculation. Images are representative of results from three samples with multiple fields of view for each sample. Data are representative of three mice. **g** Schematic of the orthotopic GL261 glioma model. **h** Kaplan–Meier survival curves of mice in (**g**) (*n* = 6 for T-Ctr group, *n* = 7 for T-αFGL2 group), log-rank test. The experiments were repeated twice with similar results.

To test the efficacy of T-αFGL2 therapy in vivo, we first validated expression of FGL2 in mouse glioma tissue. Brain tissue samples from DBT glioma-bearing mice (an immunocompetent syngeneic mouse glioma model) were cryosectioned and stained with FGL2 mAb-clone #4. As shown in Fig. 2a, both glioma cells and surrounding stroma positively stained for FGL2. Next, DBT tumor-bearing Balb/c mice were used to evaluate the antitumor effects of T-αFGL2. Mice were implanted with glioma cells and then treated with the standard chemotherapy agent temozolomide on days 3–5 to simulate standard care, and then T-Ctr or T-αFGL2 cells were injected via the tail vein on days 6 and 13 after glioma cell implantation (Fig. 2b). DBT is a very aggressive glioma, and most DBT-bearing mice in the no-treatment and T-Ctr groups died within 3 weeks. In contrast, T-αFGL2 treatment effectively suppressed DBT glioma growth, and gliomas were eliminated in about 30% of the T-αFGL2–treated mice. These mice remained glioma free for up to 70 days before being used for a rechallenge study. In contrast, gliomas progressed rapidly in T-Ctr–treated mice (Fig. 2c–f).

To confirm the efficacy of T-αFGL2 treatment, a second glioma model was used, consisting of GL261 glioma cells inoculated into the brains of immunocompetent mice. As shown in Fig. 2g, h, compared with T-Ctr, T-αFGL2 treatment suppressed glioma growth and extended mouse survival in this model as well. Overall, we conclude that T-αFGL2 inhibits the growth of glioma tumors in syngeneic murine models.

### T-αFGL2 treatment induces formation of tumor-specific CD8^+^ T_RM_-like cells in the brain

**Fig. 3:**
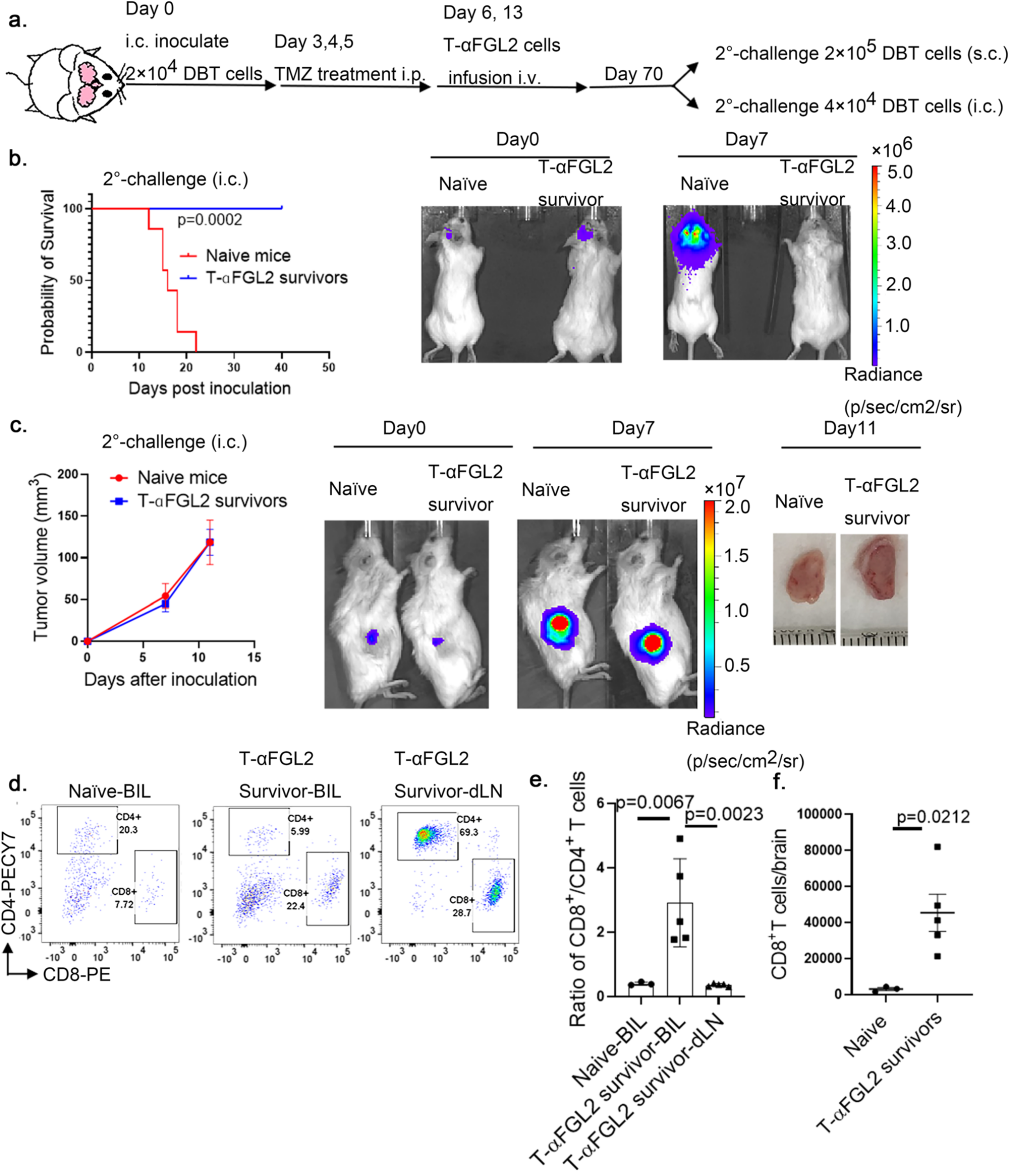
T-αFGL2 treatment induced brain-resident tumor-specific memory T cells. **a** Schematic of rechallenge with tumor cells. On day 70 after first tumor cell inoculation, T-αFGL2–treated survivors bearing orthotopic DBT gliomas were rechallenged with DBT cells injected either subcutaneously (s.c.) or intracranially (i.c.). **b** Kaplan–Meier survival curves (left) and representative bioluminescence images (right) of mice in (**a**) on day 0 and day 7 after second tumor cell inoculation (i.c.) (*n* = 6 mice). ***P = 0.0005, log-rank test. **c** Tumor volume (left) and representative bioluminescence images (middle) of mice on day 0 and day 7 after second tumor cell inoculation (s.c.), and representative tumors (right) collected on day 11 after second tumor cell inoculation (s.c.) from the flanks of Balb/c mice (*n* = 3 mice/group; data are mean ± SD). **d** Representative flow cytometry plots depicting increase of CD8^+^ T cells in the brains (BIL) of long-term survivors treated with T-αFGL2 (T-αFGL2 survivor). LN lymph node. **e** Ratio of CD8^+^ T cells to CD4^+^ T cells in the brains of naïve mice and T-αFGL2 survivors and the LNs of T-αFGL2 survivors. Data are mean ± SD, one-way ANOVA with Tukey’s test for comparing multiple treatments. **f** CD8^+^ T cell numbers in the brains of naïve mice and T-αFGL2 survivors (*n* = 3 naïve mice, *n* = 5 T-αFGL2 survivors; data are mean ± SD), two-tailed *t*-test. The experiments were repeated three times with similar results.

We next evaluated whether long-term survivors that had been treated with T-αFGL2 cells developed memory T cells that were reactive to tumor cells. The T-αFGL2–treated survivors were rechallenged with an intracranial (i.c.) implantation of DBT cells (Fig. 3a). The rechallenge DBT cells were cleared within 7 days, based on bioluminescence, in the T-αFGL2–treated survivors (Fig. 3b). Local re-exposure to DBT cells induced a rapid, more than 18-fold increase in the number of CD8^+^ T cells in the brains of T-αFGL2–treated survivors compared to the number in naïve brains (Fig. 3d, f). To investigate the tumor specificity of the generated memory T cells, we rechallenged DBT tumor–rejecting mice that had received T-αFGL2 treatment with 4T1 breast cancer cells (i.c.), which rapidly develop tumors in naïve Balb/c mice. The DBT-rejecting memory T cells failed to protect mice from 4T1 breast cancer cell challenge (Supplementary Fig. 4a). This rapid and intense T-cell reactivity to DBT cells, but not to 4T1 cells, confirmed that tumor-specific memory CD8^+^ T cells had developed in the brains of T-αFGL2–treated survivors.

To determine whether these tumor-specific memory CD8^+^ T cells stayed in the vicinity of the tumor (i.e., in the brain) or migrated throughout the body, we implanted DBT cells subcutaneously into the flanks of T-αFGL2 survivors and naïve mice (Fig. 3a). Interestingly, both groups of mice developed tumors under the skin (Fig. 3c), suggesting that the tumor-specific memory CD8^+^ T cells in T-αFGL2-treated survivors were restricted to the brain. To confirm that tumor-reactive CD8^+^ T cells only existed in the brain, we assessed T cells from the brains and draining lymph nodes (dLNs) of naïve mice and T-αFGL2-treated survivors 7 days after the rechallenge with DBT cells. As shown in Fig. 3d, e, the ratio of CD8^+^ T cells to CD4^+^ T cells in the brain was up to eightfold higher in T-αFGL2-treated survivors than in naïve mice. Moreover, the ratio of CD8^+^ to CD4^+^ T cells was ninefold higher in the brains than in the dLNs of T-αFGL2-treated survivors, suggesting that CD8^+^ T cells, but not CD4^+^ T cells, were the primary memory T cells controlling glioma cell growth, and that these CD8^+^ T cells were only resident in the brain. Taken together, these data strongly indicate that T-αFGL2 treatment induced development of brain-resident tumor-specific CD8^+^ T_RM_-like cells.

### CD8^+^ T_RM_-like cells undergo recall expansion and reject glioma cells when transplanted into naïve brains

**Fig. 4:**
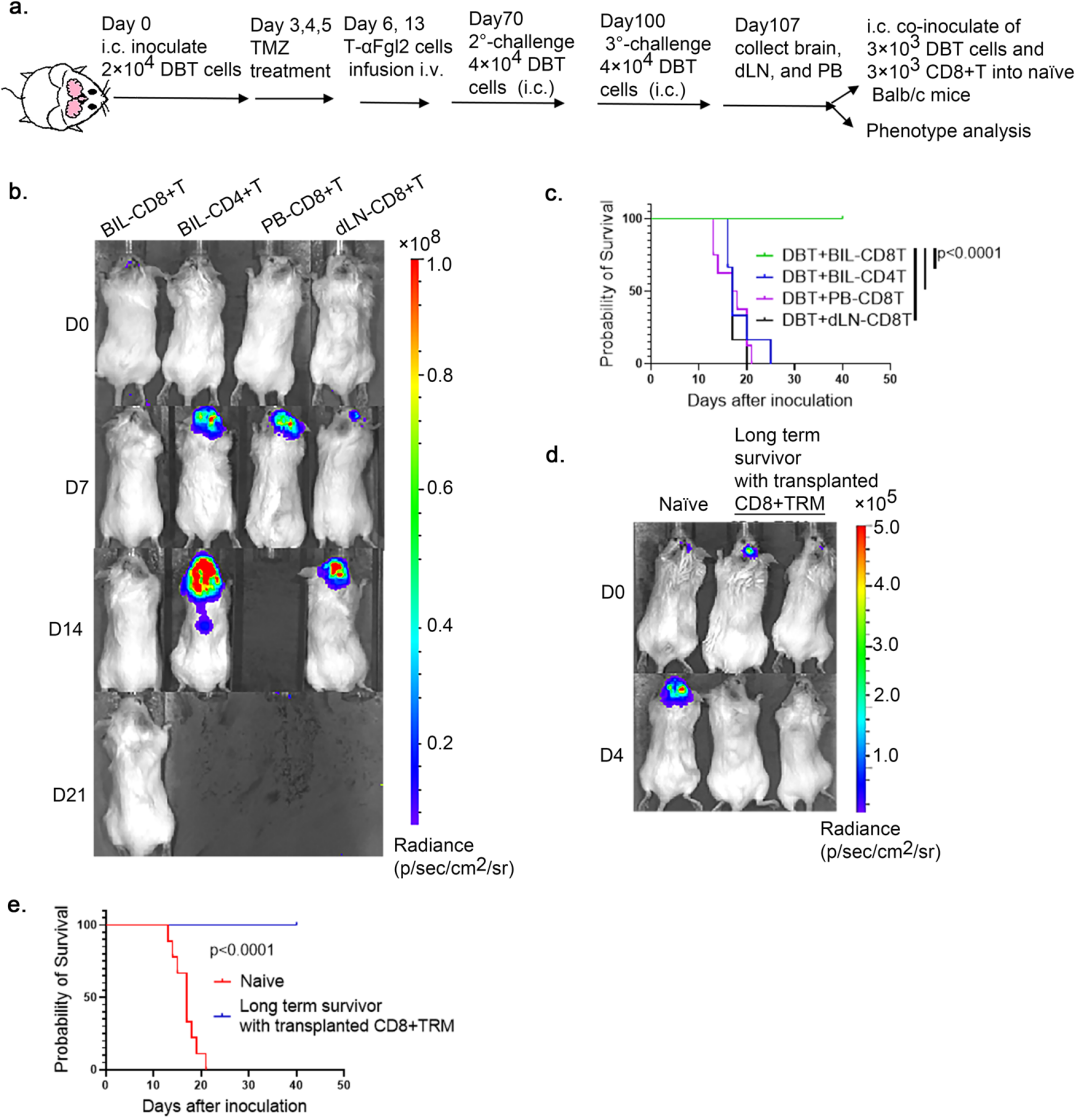
T_RM_-like cells can be adoptively transferred. **a** Schematic of experimental design. On days 70 and 100 after the first tumor cell inoculation, T-αFGL2-treated survivors were rechallenged with DBT tumor cells intracranially (i.c.). On day 7 after the third challenge (day 100) with tumor cells, the mice were euthanized, and their brains, draining lymph nodes (dLN), and peripheral blood (PB) were collected. **b** Representative bioluminescence images of naïve Balb/c mice coinoculated i.c. with 3 × 10^3^ DBT glioma cells and 3 × 10^3^ T cells. Images show gliomas in mice coinoculated with CD8^+^ T cells in the brain (BIL-CD8^+^T), CD4^+^ T cells in the brain (BIL-CD4^+^T), CD8^+^ T cells in peripheral blood (PB-CD8^+^T), or CD8^+^ T cells in draining lymph nodes (dLN-CD8^+^T). CD8^+^ T cells and CD4^+^ T cells were sorted by flow cytometry on day 7 after the third challenge in T-αFGL2–treated survivors. **c** Kaplan–Meier survival curves for mice in (**b**) (*n* = 9 in BIL-CD8^+^T group, *n* = 6 in BIL-CD4^+^T and dLN-CD8^+^T groups, *n* = 8 in PB-CD8^+^T group), log-rank test. The experiments were repeated 3 times with similar results. **d**, Representative bioluminescence images of naïve Balb/c mice and mice bearing transplanted BIL-CD8^+^T cells on days 0 and 4 after i.c. rechallenge with DBT cells on day 30 after BIL-CD8^+^T cell transplantation. **e** Kaplan–Meier survival curves of mice in (**d**) (*n* = 9 mice/group), log-rank test. Data shown are representative of three independent experiments.

To validate that CD8^+^ T cells in the brains of T-αFGL2-treated survivors were CD8^+^ T_RM_ cells, CD8^+^ T cells from the brains, dLNs, and peripheral blood (PB) of T-αFGL2-treated survivors were sorted on day 7 after tumor cell implantation and adoptively transplanted along with DBT cells directly into the brains (i.c.) of naïve recipient mice (Fig. 4a). In contrast to both dLN and PB CD8^+^ T cells, which failed to mount a recall response, brain CD8^+^ T cells underwent expansion even when reseeded in the brain tissue at low numbers (3000 cells) (Fig. 4b, c), confirming that the CD8^+^ T cells in the brains of T-αFGL2-treated survivors are bona fide T_RM_ cells. As most of the CD8^+^ T cells in tumor-experienced brains were CD44^+^ memory T cells, while the CD8^+^ T cells in peripheral tissue were not, we then compared the antitumor effect of CD44^+^CD8^+^ T cells in brain, dLNs, peripheral LNs, and PB to validate the results. Consistent with the CD8^+^ T cell data, the CD44^+^CD8^+^ T cells in peripheral tissue did not provide protection against tumor cells in vivo (Supplementary Fig. 4b, c). Similar results were found in the GL261 model (Supplementary Fig. 4d). To determine whether CD4^+^ T cells in the brain behaved similarly to the CD8^+^ T cells, we sorted CD4^+^ and CD8^+^ T cells from the brains of T-αFGL2-treated survivors and then co-implanted them with DBT cells into the brains of naïve recipient mice. As shown in Fig. 4b, c, CD4^+^ T cells did not have the same tumor cell-eliminating capacity as CD8^+^ T cells, confirming that the induced brain-resident CD8^+^ T cells, but not CD4^+^ T cells, provide immune surveillance of the previously encountered tumor antigen.

**Fig. 5:**
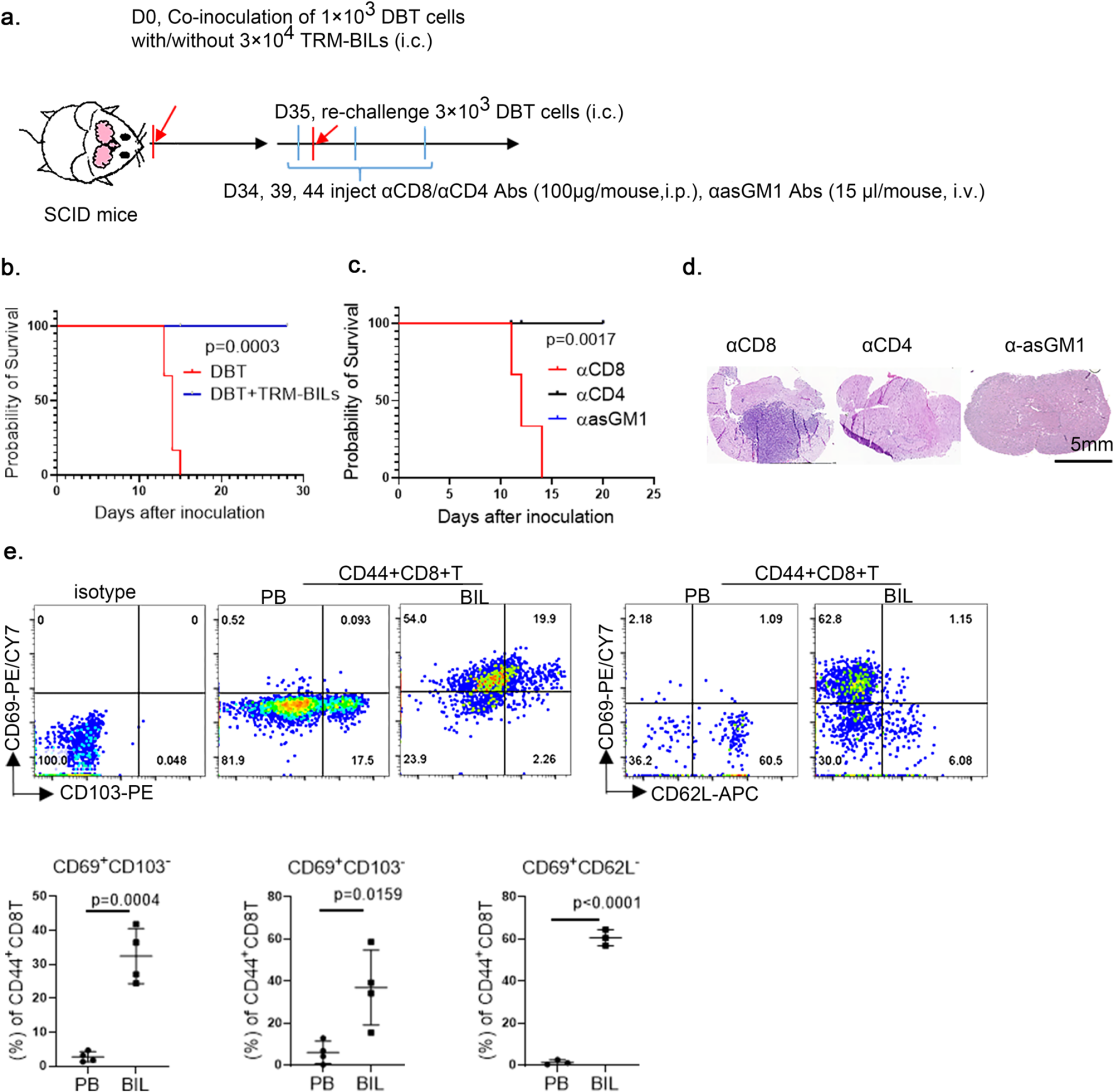
T_RM_-like cells were CD8^+^T cells and exhibited T_RM_ phenotypes. **a** Schematic of experimental design. On day 7 after the third tumor cell rechallenge, 3 × 10^4^ T_RM-_containing brain-infiltrating lymphocytes (T_RM_-BIL) from T-αFGL2–treated survivors were sorted by flow cytometry and co-inoculated i.c. along with 3 × 10^3^ DBT cells into naïve SCID mice; 35 days after transplantation, the SCID mice bearing transplanted T_RM_-BILs were rechallenged with 3 × 10^3^ DBT cells i.c., combined with antibodies blocking CD8, CD4, or asGM1 i.p. **b** Kaplan-Meier survival curves of mice in (**a**) (*n* = 6 mice/group), log-rank test. **c** Kaplan–Meier survival curves of mice treated with anti-CD8, anti-CD4, or anti-asGM1 antibodies in (**a**) (*n* = 3 mice/group), log-rank test. The experiments were repeated twice with similar results. **d** Representative H&E staining of brains from **c** collected on day 14 after tumor cell rechallenge. Similar observations were made in three mice per group and representative images are shown. **e** Representative flow cytometry plots and graph showing ratio of CD69^+^CD103^+^ T cells, CD69^+^CD103^−^ T cells, and CD69^+^CD62L^−^ T cells in brain and PB of T-αFgl2-treated survivors (*n* = 4 for detection of CD69^+^CD103^+^ T cells and CD69^+^CD103^−^ T cells, *n* = 3 for detection of CD69^+^CD62L^−^ T cells; data represent mean ± SD), two-tailed *t*-test. The experiments were repeated twice with similar results.

To further investigate whether the adoptively transplanted CD8^+^ T_RM_ cells could survive and remain in the brains of naïve recipient mice, we subsequently challenged the recipient mice with glioma cells on day 40 after adoptive CD8^+^ T cell transplantation. We observed that the tumor cells were rejected in the recipient mice (Fig. 4d, e). These findings show that when CD8^+^ T_RM_ cells were successfully transplanted into naïve brains, naïve brains could be transformed to become tumor-rejecting brains. To further validate the function of transplanted CD8^+^ T_RM_ cells and exclude the effect of host T cells, we transplanted brain-infiltrating lymphocytes (BILs) from brains bearing T_RM_ cells (T_RM_-BILs) into the brains of naïve immunodeficient SCID mice and challenged these mice 35 days after the transplantation (Fig. 5a). Similar to the results obtained for the parental T_RM_-bearing mice, the brains of these T_RM_-BIL recipient SCID mice showed antitumor capacity (Fig. 5a, b). To verify that the transplanted CD8^+^ T cells were responsible for this glioma-protective capacity, we challenged these SCID survivors with glioma cells combined with αCD8, αCD4, or αAsialo-GM1 antibodies to deplete CD8^+^ T cells, CD4^+^ T cells, and natural killer (NK) cells, respectively. Only depleting CD8^+^ T cells, not CD4^+^ T cells or NK cells, impaired the antitumor protection (Fig. 5c, d). Thus, CD8^+^ T_RM_-like cells in the brains of T-αFGL2-treated survivors, which fulfill both memory and reactive functions against tumor cells, are tumor-specific brain CD8^+^ T_RM_ cells. Notably, these CD8^+^ T_RM_ cells can be adoptively transferred into naïve brains with or without host T cells.

### CD8^+^ T_RM_ cells establish a classical T_RM_ phenotype

To determine whether these CD8^+^ T_RM_-like cells have a classical T_RM_ phenotype, expression of CD69, CD103, and CD62L was evaluated^2,11,24^. To verify that the isolated BILs-CD8^+^ T cells were brain restricted, we performed intravenous injection of a CD8β antibody and found that over 90% of BIL-CD8^+^ T cells were noncirculating brain-resident T cells (Supplementary Fig. 5a). When compared with CD44^+^CD8^+^ T cells in PB, the CD44^+^CD8^+^ T cells in T_RM_-bearing brains were CD69^+^ (either CD103^+^ or CD103^−^) and CD62L^−^ (Fig. 5e). Similar results were found for CD4^+^ T cells (Supplementary Fig. 5b). These findings show that CD8^+^ T_RM_-like cells in brains established a classical T_RM_ phenotype of CD69^+^CD62L^−^. Together, both the function and phenotype of the CD8^+^ T_RM_-like cells in brains of T-αFGL2-treated survivors further validated that these cells are tumor-specific brain CD8^+^ T_RM_ cells.

### The function of CD8^+^ T_RM_ cells is TCR-MHC-I–dependent

**Fig. 6:**
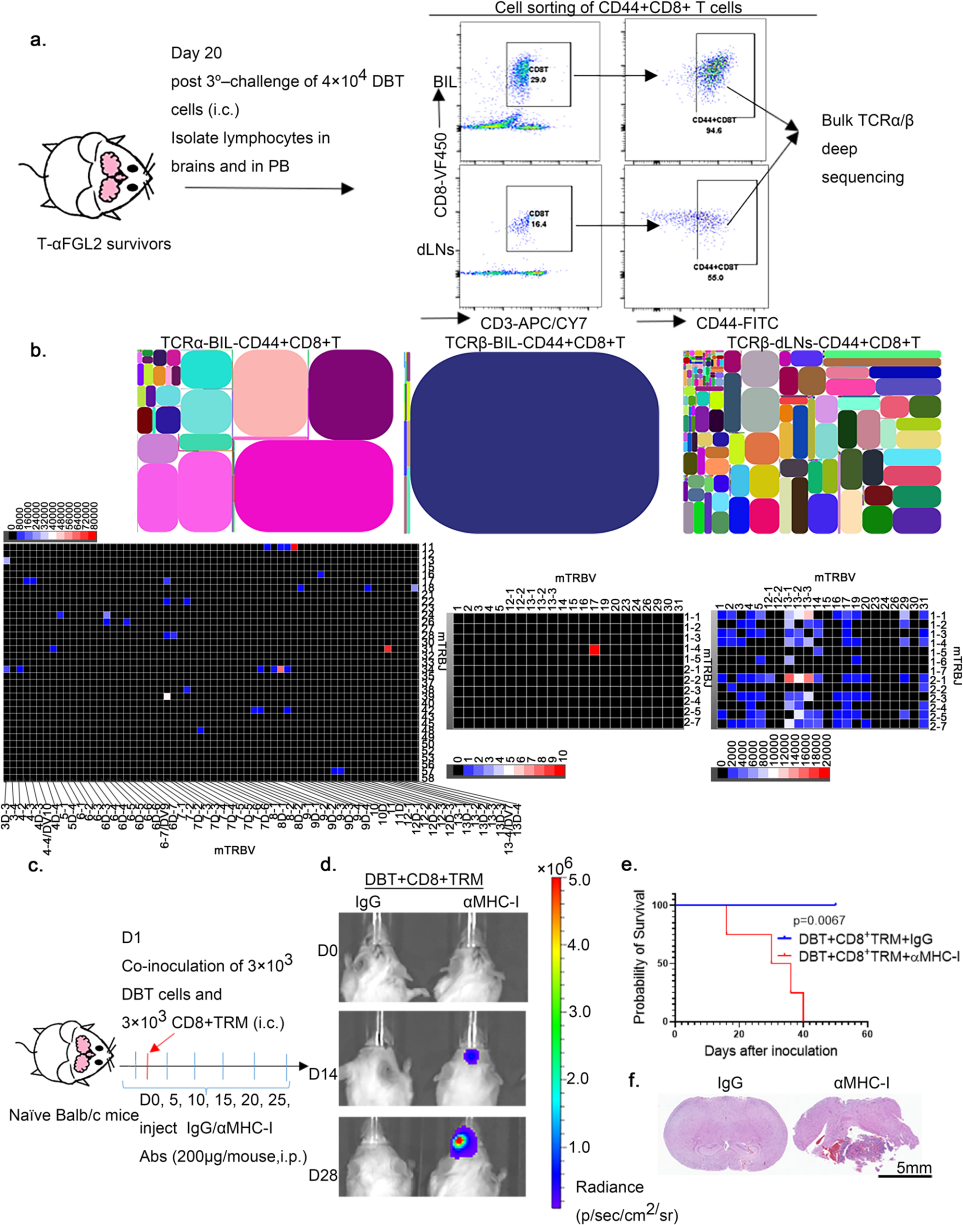
T_RM_ cells showed the presence and expansion of unique T cell clones. **a** Schematic of TCRα/β deep sequencing of CD8^+^ T cells from the brains and draining lymph nodes (dLNs) of T-αFGL2-treated survivors. Cells were sorted via flow cytometry on day 20 after the third challenge with intracranially (i.c.) injected DBT tumor cells. **b** Representative tree maps (top row) of TCRα-T_RM_ -CD8^+^T, TCRβ -T_RM_ -CD8^+^T, and TCRβ-dLNs-CD8^+^T cell clones. Each spot represents a unique entry: V-J-CDR3, and the size of a spot denotes its relative frequency; 2D map of V and J usage of TCRα-T_RM_ -CD8^+^T, TCRβ-T_RM_ -CD8^+^T, and TCRβ-dLNs-CD8^+^T cell clones (bottom row). The experiments were repeated twice with similar results. **c**, Schematic of experimental design. On day 1, 3 × 10^4^ CD8^+^T_RM_ cells and 3 × 10^3^ DBT cells were coinoculated i.c. into naïve Balb/c mice; on days 0, 5, 10, 15, 20, and 25, the mice were treated with IgG or MHC-I blocking antibodies (100 µg/mouse, i.p.). **d** Representative bioluminescence images of Balb/c mice on days 0, 14, and 28 after i.c. coinoculation with CD8^+^T_RM_ and DBT cells. **e** Kaplan–Meier survival curves of mice in (**f**) (*n* = 4 mice/group), log-rank test. The experiments were carried out twice with similar results. **f** Representative H&E staining of brains from (**d**) collected on day 40 after transplantation. Similar observations were made in three mice per group and representative images are shown.

Since TCR is generated through random rearrangement of genomic V(D)J segments and is the mediator of antigen recognition and binding by T cells, we next evaluated whether CD8^+^ T_RM_ cells displayed a unique TCR repertoire that was distinct from that found in the dLNs. To this end, CD44^+^CD8^+^ T (memory CD8^+^ T) cells were sorted via flow cytometry from the brains and dLNs of T-αFGL2-treated survivors on day 20 after the third challenge with DBT cells, followed by TCRα and TCRβ deep sequencing (Fig. 6a). The most abundant T cell clones—those with a frequency of more than 5%—in T_RM_-bearing brains constituted more than 60% of the total TCRα and TCRβ repertoire, whereas no T cell clones with a frequency of more than 5% were found in the TCRβ repertoire of CD44^+^CD8^+^ T cells in the dLNs (Supplementary Fig. 5c). To further characterize the TCR repertoires of T_RM_ cells and dLN-CD44^+^CD8^+^ T cells, the sequences of complementarity determining region 3 (CDR3), which encompasses the V(D)J recombination junctions and encodes the vast majority of TCR variation, were analyzed. All of the top 10 dominant CDR3 sequences in T_RM_ cells encompassed the V/J recombination, but each dominant CDR3 sequence in dLN-CD44^+^CD8^+^ T cells encompassed a unique V/J recombination (Fig. 6b and Supplementary Fig. 5d). Analysis of the V and J domain usage showed that in one of the T-αFGL2-treated survivors, the most dominant clone of TCRβ in T_RM_ cells was grouped by V17/J1-4, which was absent in dLNs (Fig. 6b). These data not only showed the presence and expansion of unique T cell clones, but also that there was no overlap between the highly occupied TCR clone in T_RM_ cells relative to the TCR clone in dLN-CD44^+^CD8^+^ T cells. Interestingly, each T-αFGL2–treated survivor mouse bore different T_RM_ clones against different antigens. These data suggested that these highly expanded TCR clones of CD8^+^ T_RM_ cells were associated with the rapid and robust response of T_RM_ cells against tumor cells.

To verify that the robust response of CD8^+^ T_RM_ cells against tumor cells is associated with the interaction between expanded TCR and MHC-I, MHC-I was blocked in vivo using an αMHC-I antibody when CD8^+^ T_RM_ cells were transplanted into naïve mice. Blocking MHC-I abolished the antitumor efficacy of the transplanted CD8^+^ T_RM_ cells (Fig. 6c–f), demonstrating that a TCR-MHC-I interaction is required for the proper function of CD8^+^ T_RM_ cells in vivo.

To clarify whether these de facto brain-resident CD8^+^ T_RM_ cells were generated from endogenous host or exogenously infused T cells, CD90.1^+^ T cells were transduced with an αFGL2-scFv-His-tag lentivirus and intravenously infused into tumor-bearing CD90.2^+^ recipient mice. After T cell infusion, the number of exogenous CD90.1^+^ T-αFGL2 cells in the brains increased from day 1 to day 4 but then began to decline (Supplementary Fig. 6a–c). The number of CD90.1^+^ T-αFGL2 cells in brains declined dramatically on day 7 and became almost undetectable on day 12 (Supplementary Fig. 6b). Based on these in vivo kinetics, a twice-weekly schedule was established to boost tumor-specific T_RM_ induction. Even with this frequent dosing, the exogenous T-αFGL2 cells became undetectable in the T_RM_-bearing brains by flow cytometry (Supplementary Fig. 6e), immunohistochemistry staining (Supplementary Fig. 6f), and the extremely sensitive assay droplet digital PCR (Supplementary Fig. 6g). That is, only host T cells (CD90.2^+^) were found in these T_RM_-bearing brains (Supplementary Fig. 6e–g), suggesting the T_RM_ cells had been programmed from host T cells. Cumulatively, these data showed that infused T-αFGL2 cells fostered the programming of endogenous T cells into CD8^+^ T_RM_ cells, but they did not themselves form the population of brain-resident CD8^+^ T_RM_ cells.

### T-αFGL2 treatment-induced CD69 expression on CD8^+^ memory T cells is essential for CD8^+^ T_RM_ cell formation

**Fig. 7:**
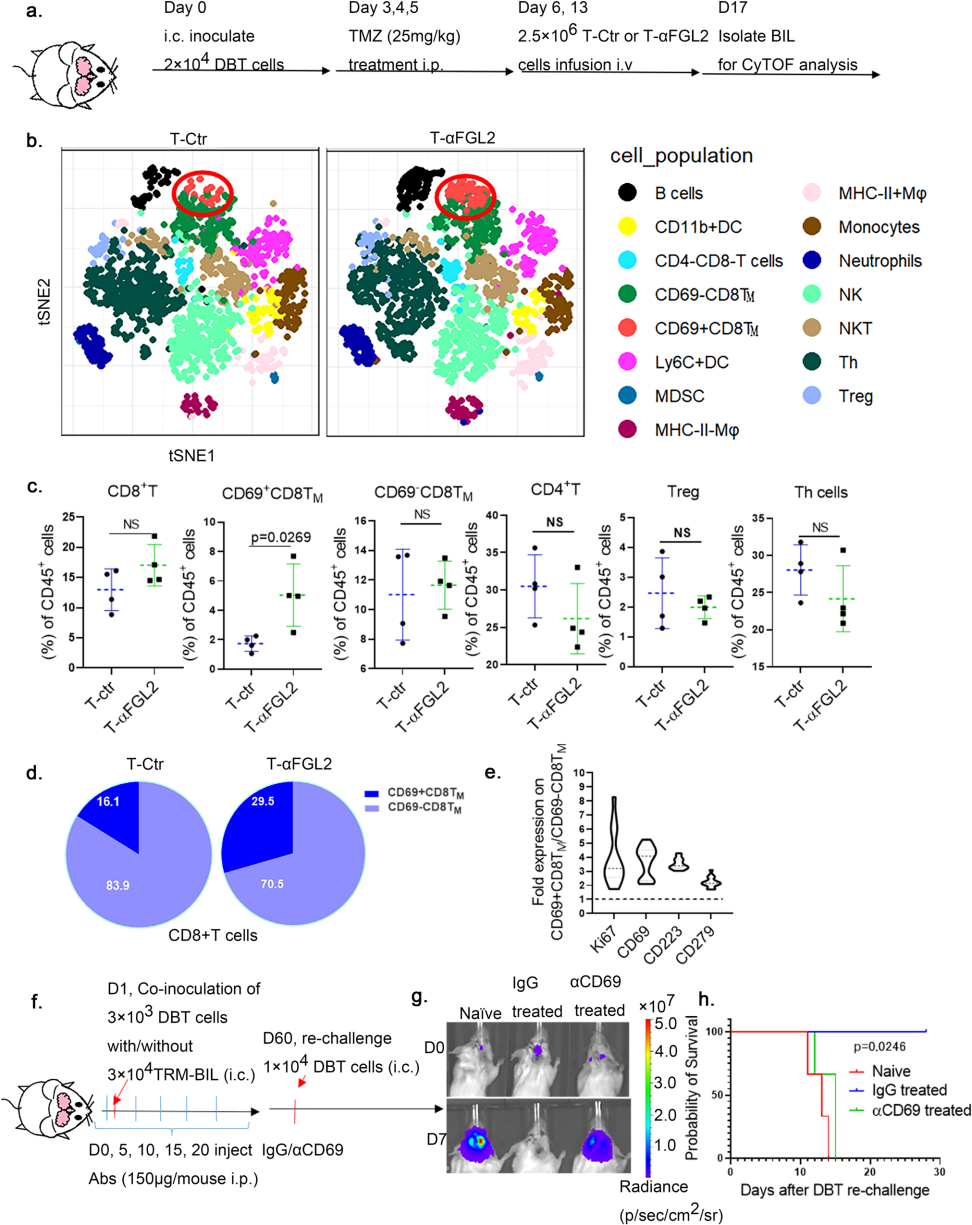
T-αFGL2 treatment increased the CD69^+^CD8^+^T_M_ cell subset. **a** Schematic of the experimental design. Four days after the second infusion of T-Ctr or T-αFGL2 cells, brains were collected to isolate brain-infiltrating lymphocytes (BILs), which were then stained with antibodies conjugated to metal isotopes. Single-cell mass cytometry (CyTOF) data were clustered to identify common populations across the treatment groups (*n* = 4 mice per group). The experiment was carried out once. **b** Analysis of CD45^+^ cells from the brain, colored by relative expression of CyTOF markers. Cell populations are indicated on the right. **c** Frequencies of total CD8^+^ T cell population and subsets of CD8^+^ T cells and CD4^+^ T cells (*n* = 4 mice per group; data represent mean ± SD), two-tailed *t*-test. **d** Composition of the CD8^+^ T cell compartment in T-Ctr and T-αFGL2-treated DBT-bearing mice showing increased frequency of CD69^+^CD8^+^ T_M_ cells in the T-αFGL2 group. **e**, Fold expression of Ki67, CD69, CD223, and CD279 on the CD69^+^CD8^+^T_M_ subset and the CD69^−^CD8^+^T_EM_ subset. **f** Schematic of experimental design. On day 1, 3 × 10^4^ CD8^+^ T_RM_ cells and 3 × 10^3^ DBT cells were coinoculated i.c. into naïve Balb/c mice; on days 0, 5, 10, 15, and 20, the mice were treated with either IgG or CD69 blocking antibodies (150 μg/mouse, i.p.); on day 60, Balb/c mice bearing transplanted CD8^+^T_RM_ were rechallenged with 1 × 10^4^ DBT cells (i.c.). **g** Representative bioluminescence images of Balb/c mice on days 0 and 7 after i.c. rechallenge with DBT cells in (**f**). Data are representative of two independent experiments. **h** Kaplan–Meier survival curves of mice in (**f**) (*n* = 3 mice/group), log-rank test. The experiments were carried out twice with similar results.

To understand the cellular mechanisms by which the FGL2-blocking scFv induces the generation of CD8^+^ T_RM_ cells, on day 4 after the second T cell infusion, high-dimensional profiling of BILs was performed using time-of-flight mass cytometry (CyTOF) with a panel of 37 antibodies including various immune cell lineage markers (Fig. 7a). T-distributed stochastic neighbor embedding (tSNE) analysis of the CyTOF data divided BILs into 15 immune cell populations (Fig. 7b). As CD8^+^ T cells are the primary functional cells rejecting tumor cells, as shown in our transplant study (Fig. 4b), we focused on these cell populations. We found that the CD8^+^ T cell population was composed of two subpopulations: CD69^+^CD8^+^ memory T cells (CD69^+^CD8^+^ T_M_) and CD69^−^CD8^+^ memory T cells (CD69^−^CD8^+^ T_M_) (Fig. 7b). Notably, the subset of CD69^+^CD8^+^ T_M_ cells was significantly larger in mice that underwent T-αFGL2 treatment than in those that received T-Ctr treatment (Fig. 7c, d). Indeed, CD69 has been reported to help in the retention of memory T cells in peripheral tissue through inhibiting expression of the S1P receptor, which can promote T cell circulation into the blood. As such, high expression of CD69 on T cells is an indicator of T_RM_ cells^24^. To further determine the phenotype of these CD69^+^CD8^+^ T_M_ cells, we compared their expression of T cell exhaustion markers with that of CD69^−^CD8^+^ T_M_ cells. As shown in Fig. 7e, CD69^+^CD8^+^ T_M_ cells had higher levels of Ki67, CD223 (LAG3), and CD279 (PD-1) than did CD69^−^CD8^+^ T_M_ cells, suggesting these CD69^+^CD8^+^ T_M_ cells were highly proliferating tumor-reactive T cells. Also, this CD69^Hi^PD-1^Hi^LAG3^Hi^ phenotype of highly proliferative CD69^+^CD8^+^ T_M_ cells has been reported to be the most prominent in cells with T_RM_ characteristics in different kinds of tissue, including the lung^16,25,26^, breast^3^, and skin^7^. These data indicate that T-αFGL2 treatment increased proliferating CD69^+^CD8^+^ T_M_ cell subsets with T_RM_ characteristics, which may promote the transformation of these CD69^+^CD8^+^ T_M_ cells into T_RM_ cells in the brain. To further validate the biological function of CD69 on CD8^+^ T_RM_ cells, CD69 was blocked in vivo by an αCD69 antibody. When the CD69^+^CD8^+^ T_RM_ cells and tumor cells were transplanted into naïve mice i.c., the αCD69 antibody treatment did not disrupt the antitumor efficacy of CD8^+^ T_RM_ cells. However, when these mice were rechallenged with glioma cells i.c. on day 60 after the transplantation, the mice treated with αCD69 antibody lost their tumor-rejecting capacity (Fig. 7f, h) indicating that CD69 does not affect the executive function of T_RM_ cells but is required for their prolonged residence and function in brains.

The analysis of the other immune populations such as helper T cells, regulatory T cells, DCs, macrophages, monocytes, and neutrophils did not reveal any significant difference between the T-αFGL2 and T-Ctr groups (Supplementary Fig. 7), suggesting that the antitumor effect induced by T-αFGL2 treatment differs from that of antibody therapy and may work mainly through regulating CD69^+^CD8^+^ T_M_ cells.

### T-αFGL2-induced CD8^+^ T_RM_ cell formation is associated with the CXCL9/10-CXCR3 axis

**Fig. 8:**
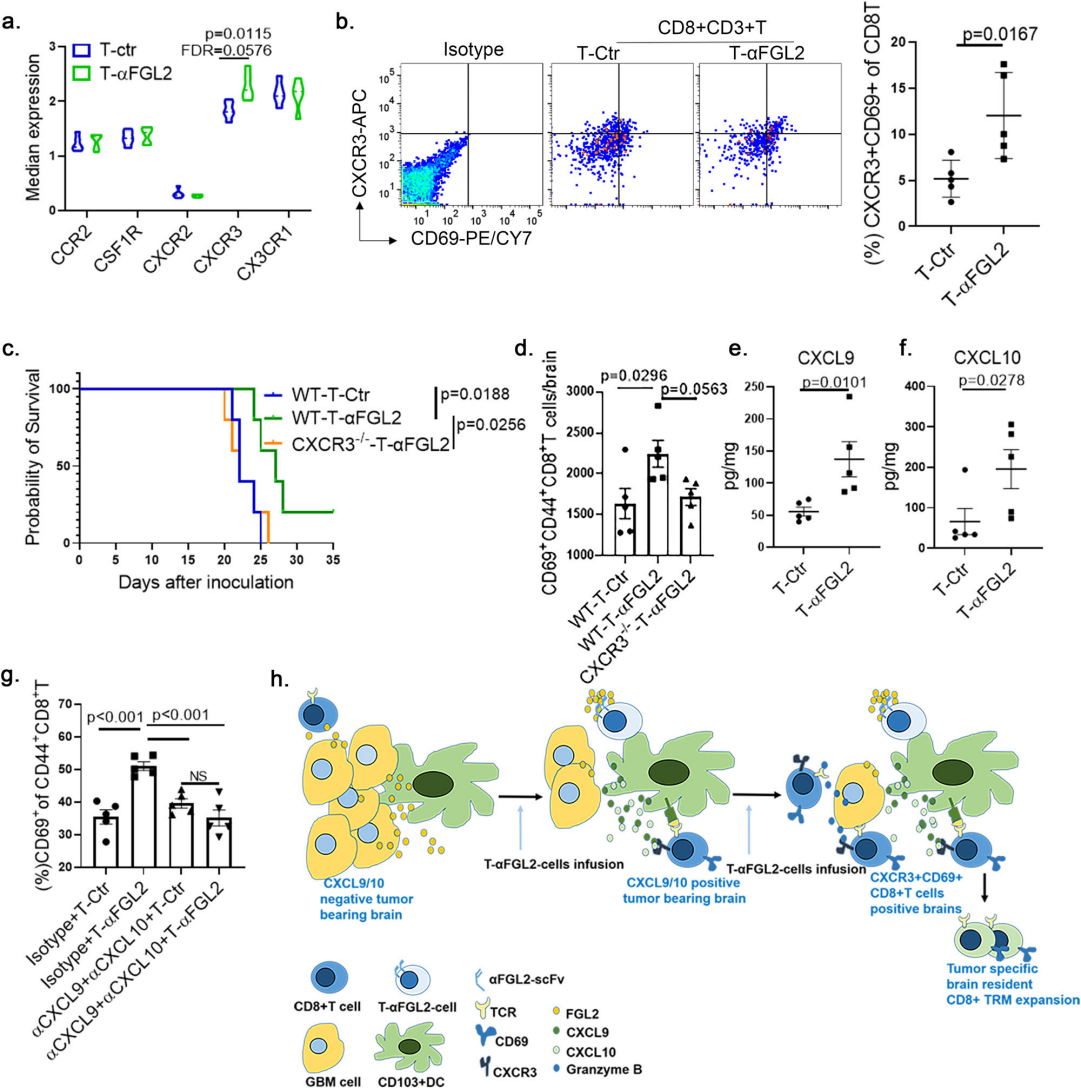
T-αFGL2-induced CD69^+^CD8^+^T_M_ cells were associated with CXCL9/10-CXCR3 axis. **a** Expression levels of CCR2, CSF1R, CXCR2, CXCR3, and CX3CR1 on CD69^+^CD8^+^T_M_ cell populations; CyTOF analysis was conducted on day 2–4 after the second T cell treatment. (*n* = 5 mice per group; data represent mean ± SD), *p* value from two-tailed *t*-test, FDR false discovery rate. **b** Representative flow cytometry plots and graphs showing that T-αFGL2 treatment increased the proportion of CXCR3^+^CD69^+^CD8^+^T cells among total CD8^+^T cells in glioma-bearing brains (*n* = 5 mice/group; data represent mean ± SD), two-tailed *t*-test. **c** Kaplan–Meier survival curves of GL261-bearing WT mice and CXCR3^−/^^−^ mice treated with T-Ctr or T-αFGL2 (*n* = 5 mice/group), log-rank test. **d** Number of CD69^+^CD8^+^T_M_ cells per GL261-bearing brain on day 5–7 after the second T cell infusion (*n* = 5 per group; data represent mean ± SEM), one-way ANOVA with Dunnett’s test for comparing multiple treatments. **e**, **f** Quantitative analysis of CXCL9 and CXCL10 protein levels in DBT tumors from mice 4–6 days after the second infusion of T-Ctr or T-αFGL2 cells (*n* = 5 per group; data represent mean ± SEM), two-way *t*-test. **g** Percentages of CD69^+^ cells among CD44^+^CD8^+^T cells (*n* = 5 per group; data represent mean ± SEM), one-way ANOVA with Dunnett’s test for comparing multiple treatments. NS not significant. **h** Schematic illustration of cellular and molecular events underlying T-αFGL2–induced tumor-specific brain-resident CD8^+^T_RM_ cells. T-αFGL2 cells block FGL2 in the tumor microenvironment, resulting in CXCL9/10 induction. The CXCL9/10-CXCR3 engagement boosts recruitment of CXCR3^+^CD69^+^CD8^+^T cells, which are candidate tumor-specific brain-resident CD8^+^T_RM_ cells.

To further understand the molecular mechanism by which the FGL2-blocking scFv induces CD69^+^CD8^+^ T_M_ cell generation, we analyzed CyTOF data for chemokine receptors (i.e., CCR2, CXCR3, CXCR2, and CX3CR1) that may affect T cell infiltration and recruitment to tumor sites. Intriguingly, the T-αFGL2 treatment increased CXCR3 expression on CD69^+^CD8^+^ T_M_ cells in glioma-bearing brains compared with T-Ctr treatment (Fig. 8a). Flow cytometry data also verified that T-αFGL2 treatment, compared with T-Ctr, increased the proportion of CXCR3^+^CD69^+^CD8^+^ T cells among total CD8^+^ T cells in glioma-bearing brains (Fig. 8b), indicating that increased CXCR3 expression on CD8^+^ T cells may play a role in T cell recruitment and in mediating T-αFGL2-induced CD69^+^CD8^+^ T_RM_ cell formation. To validate this idea, we compared the antitumor efficacy of T-αFGL2 in treating glioma-bearing wild-type (WT) mice and CXCR3-deficient (CXCR3^-/-^) mice. T-αFGL2 treatment did not show a protective effect in the CXCR3^−^^/−^ mice but did in WT mice (Fig. 8c). Furthermore, the CD69^+^CD8^+^ T_M_ population was reduced in CXCR3^−/−^ mice compared with WT mice (Fig. 8d), suggesting that CXCR3 plays a critical role in mediating T-αFGL2-induced CD8^+^ T cell recruitment and subsequent CD69^+^CD8^+^ T_M_ cell and CD8^+^ T_RM_ cell formation.

To characterize how the CXCR3 chemokine system mediates antitumor responses to T-αFGL2 treatment, the expression of the CXCR3 chemokine ligands CXCL9 and CXCL10 was evaluated. Protein levels of CXCL9 and CXCL10 were markedly higher in tumor-bearing brains after T-αFGL2 treatment compared to T-Ctr treatment (Fig. 8e, f). To determine the roles of CXCL9 and CXCL10 in T-αFGL2-induced tumor rejection and CD8^+^ T_RM_ cell formation, αCXCL9 and αCXCL10 antibodies were used to block CXCL9 and CXCL10 in vivo. Consistent with our earlier findings (Fig. 8d), the percentage of CD69^+^CD8^+^ T_M_ cells was increased upon T-αFGL2 treatment in the group treated with control IgG but not in the groups treated with αCXCL9 and αCXCL10 antibodies (Fig. 8g), indicating the functional importance of the CXCL9/10-CXCR3 axis for response to T-αFGL2 therapy and CD8^+^ T_RM_ cell formation. In sum, T-αFGL2 therapy induced tumor-reactive T cell proliferation, promoted secretion of granzyme B to control tumor progression, and increased CXCR3 and CD69 expression on CD8^+^ T_M_ cells to facilitate their brain retention, which fostered the formation of tumor-specific brain-resident CD8^+^ T_RM_ cells (Fig. 8h).

To examine the direct role of T-αFGL2 in the encounter between T cells and tumor cells, we cocultured DBT glioma cells with T-Ctr or T-αFGL2 cells for 72 h ex vivo, sorted CD8^+^ T cells via flow cytometry, and conducted next-generation sequencing of the CD8^+^ T cells. Notably, T-αFGL2 cells cocultured with glioma cells, compared with the T-Ctr cells, showed markedly diminished levels of the transcript encoding P53-induced death domain protein 1 (PIDD), which is an effector of P53-dependent apoptosis (Supplementary Fig. 8a). To confirm that FGL2 modulated PIDD expression and induced apoptosis of T cells, Western blotting and Annexin V staining were performed using WT and FGL2^−^^/−^ T cells. Both PIDD expression and the proportion of apoptotic cells were significantly lower in FGL2^−/−^ T cells than in WT T cells (Supplementary Fig. 8b, c). Similarly, in T-αFGL2 cells cocultured with FGL2-expressing DBT cells, the number of T-αFGL2 cells undergoing apoptosis was lower than that in cocultured T-Ctr cells (Supplementary Fig. 8d). To further analyze whether P53 signaling was downregulated by the FGL2-blocking scFv, Gene Set Enrichment Analysis (GSEA) was performed, revealing that the expression of genes associated with P53 signaling was lower in T-αFGL2 cells than in T-Ctr cells in the context of coculture with glioma cells (Supplementary Fig. 8e, normalized enrichment score [NES] = −2.0, *P* = 0.009). These data clarify that blocking FGL2 with scFv protected a subpopulation of T cells from apoptosis, which may favor their long-term survival in peripheral tissue.

Finally, to evaluate if T-αFGL2 directly affected T_RM_ cell formation in coculture with tumor cells, core circulation genes and core T_RM_ genes were analyzed. Interestingly, expression of the core circulation genes was significantly lower in T-αFGL2 cells than in T-Ctr cells. However, the T-αFGL2 cells showed a trend toward enrichment in core T_RM_ genes when compared with T-Ctr cells (Supplementary Fig. 8f, NES = 1.23, *P* = 0.107), suggesting that T-αFGL2 treatment does not directly transform regular T cells into brain tumor–specific T_RM_ cells. Cumulatively, these findings demonstrate that T-αFGL2 cells, when cocultured with tumor cells, decrease expression of genes associated with apoptosis and circulating memory T cells, which may favor T_RM_ formation and survival.

### T-αFGL2 treatment disrupts FcγRIIB-FGL2 interactions to boost the cytotoxic T cell response

Immune-suppressive FGL2 limits cytotoxic CD8^+^ T cell responses via FcγRIIb^27^. To evaluate whether the T cells in glioma-bearing brains express FcγRIIb, which can bind and be regulated by FGL2 in the tumor microenvironment, FcγRIIB expression was detected on T cells in DBT tumor-bearing brains. Both CD4^+^ and CD8^+^ T cells (though not all of them) expressed FcγRIIB (Supplementary Fig. 9a). Moreover, WT T cells, but not FcγRIIB^−/−^ T cells, directly bound the FGL2 anchored on the microfluidic chip via the biotin-streptavidin interaction (Supplementary Fig. 9b), verifying that T cells can directly bind FGL2 through FcγRIIB. Furthermore, the therapeutic effect of T-αFGL2 treatment was lost in FcγRIIB^−/^^−^ mice (Supplementary Fig. 9c), suggesting that FcγRIIB was regulated by FGL2. These data suggested that the T cells in this study can bind and be regulated by FGL2 in the tumor microenvironment through FcγRIIB and that T-αFGL2 treatment can disrupt this FcγRIIB-FGL2 interaction and boost the cytotoxic T cell response.

## Discussion

Increasing evidence has shown that T_RM_ cells have a promising role in the control of solid tumors^2^. Early studies showed that intravaginal human papillomavirus vaccination induced T_RM_ formation in cervicovaginal tissue resulting in the control of tumors in the genital tract^28^. Nonetheless, whether adoptive cellular therapies (ACTs) can foster T cell development into T_RM_ cells remains unknown. Our study has developed an ACT-based treatment, T-αFGL2 cell therapy, that can program endogenous T cells into tumor-specific CD8^+^ T_RM_ cells. These T_RM_ cells have a highly expanded and specific TCR repertoire. After being transplanted into the brains of either immunocompetent or T cell–deficient naïve mice, these T_RM_ cells transform the brain from one that was hospitable to tumors to one that was hostile.

Retention in the resident tissue is required for T_RM_ cells to expand and be functional^11^. One of the mechanisms of T_RM_ cell retention is adhesion to the homing tissue, which is associated with overexpression of integrin molecules such as LFA-1 (αLβ2), VLA-1(α1β1)^29^, and CD103 (αEβ7)^11,29^; binding to adhesion molecules on the endothelium; and the extracellular matrix components collagen and laminin. Another mechanism accounting for retention of T_RM_ cells in the tissue is unresponsiveness to signals that promote recirculation. Expression of S1PR1 (encoded by *S1pr1*)^30^, CD62L (encoded by *Sell*), and CCR7 (encoded by *Ccr7*)^31^ permits recirculation of memory T cells. It has been reported that CD69 suppresses memory T cells’ recirculation potential by inhibiting surface expression of the S1P receptor^24^. Indeed, most T_RM_ cells constitutively express CD69^6,10,32^. Although CD103, which binds to E-cadherin on epithelial cells, is expressed on the most-studied T_RM_ cells (those residing in epithelial tissue^29^), CD69 is more commonly used to identify T_RM_ cells in non-lymph organs. In our study, T-αFGL2, compared with T-Ctr, increased the population of CD69^+^CD8^+^ T_RM_-like cells in the brain tumor environment. The T-αFGL2 treatment induced CD8^+^ T_RM_ cells (phenotype: CD69^+^CD62L^−^), and blocking CD69 appears to disrupt the residence of these brain-resident T_RM_ cells. Altogether, the increased expression of the tissue retention molecule CD69 on CD8^+^ memory T cells in the brain helps retain these cells in the brain and promote their differentiation into brain-resident T_RM_ cells. Of course, CD69 may not act alone; GSEA data revealed that T-αFGL2 cells, when cocultured with tumor cells, exhibited decreased expression of circulation-associated genes. Apart from this, T-αFGL2 cell coculture with glioma cells also reduced expression of genes associated with the P53 signaling pathway, especially PIDD, which can protect T cells from apoptosis and lead to their long-term persistence in vivo. Altogether, T-αFGL2–induced T_RM_ cell formation in the brain may depend on both retention and survival mechanisms.

The formation of CD69^+^CD8^+^ T_RM_ cells induced by T-αFGL2 treatment is associated with the CXCL9/10-CXCR3 axis, as either absence of CXCR3 or blocking CXCL9/10 abrogated the increase in CD69^+^CD8^+^ T_M_ cells after T-αFGL2 treatment, and thus interrupted the antitumor efficacy of T-αFGL2 treatment. The notion that the CXCR3-CXCL9/10 axis promotes the generation of T_RM_ cells is consistent with previous findings that this CXCR3-CXCL9/10 axis is required for T cell recruitment into the brain^33–35^, reinvigoration of intratumoral CD8^+^ T cell responses in response to PD-1 blockade^36^, and exogenous application of CXCR3 ligands to promote T_RM_ cell formation in the epithelium of the lower female reproductive tract.

To date, most studies of T_RM_ cells have focused on infectious diseases rather than cancer. Malik *et al*.^37^ reported that melanoma antigen–specific skin-resident memory T cells are maintained in vitiligo-affected skin, but no ACT that induces tumor-specific T_RM_ cells has yet been evaluated. Here, we performed T-αFGL2 ACT in brain tumor-bearing mice and found that T-αFGL2 induced tumor-specific CD8^+^ T_RM_ cells in the brain. The phenotypes of these CD8^+^ T_RM_ cells were either CD69^+^CD103^+^ or CD69^+^CD103^−^. In addition to characterizing the phenotypes of these tumor-specific T_RM_ cells, we characterized their TCR repertoires. TCR recognizes antigens presented by the MHC on antigen-presenting cells and subsequently activates T cells and mediates the eradication of the antigen. However, the TCR repertoires of tumor-specific T_RM_ cells have not previously been characterized. From next-generation RNA sequencing, we noted that the CD8^+^ T_RM_ cells had a highly expanded TCRαβ repertoire. Moreover, no overlap of the TCR clone was found between T_RM_ cells in the brain and memory T cells in the periphery. This differs from influenza-specific lung T_RM_ cells, which maintain a wide diversity of TCR profiles^14^. Depleting MHC-I abolished the antitumor efficacy of the CD8^+^ T_RM_ cells in this study, demonstrating that a TCR-MHC-I interaction is required for the proper function of CD8^+^ T_RM_ cells in vivo. Further functional evaluation of this highly expanded TCR repertoire on T_RM_ cells will be an important part of future studies.

T_RM_ cells occupy frontline sites of infection and are positioned to respond most immediately and potently. The abundance of cells with T_RM_ cell characteristics in tumors often correlates with a favorable outcome^3,27,38,39^. Thus, promoting tumor-specific T_RM_ cell formation in tumor tissue or adoptively transferring tumor-specific T_RM_ cells into tumor sites are promising approaches for treating patients with cancer. Wakim et al.^13,40^ found that virus-specific T_RM_ cells in the brain die rapidly upon isolation from the resident tissue and fail to undergo recall expansion after adoptive transfer into the bloodstream of an antigen-challenged recipient, indicating that these cells depend on the local milieu for their function and survival. Ning and colleagues^41^ found that injection of a viral antigen peptide converted infused memory T cells to a T_RM_ phenotype in brain tumors but did not induce rejection of the tumors. Here, we were able to transfer CD8^+^ T_RM_ cells into the brains of both immunocompetent and T cell–deficient naïve mice and induce a tumor-specific reaction in the recipient mice. Our success may be explained by the following factors: (1) T_RM_ cells should be transferred into the same tissue in naïve mice where the T_RM_ cells originally resided; and (2) T_RM_ cells should be co-transferred with antigens that can activate them. Besides yielding novel insights into these tumor-specific brain-resident CD8^+^ T_RM_ cells, our study provides a valuable resource for further investigations of tumor-specific T_RM_ cell formation in the brain. Such studies will ultimately aid the development of strategies for immunotherapy of brain cancers.

## Methods

### Human samples

Healthy human tissue array sections were purchased from US Biomax, Inc (Cat#: FBN406et). Tumor tissue sections from patients newly diagnosed with primary GBM were kindly provided by Dr. Amy B. Heimberger (The University of Texas MD Anderson Cancer Center). These studies were conducted under protocol #LAB03-0687, which was approved by the Institutional Review Board of The University of Texas MD Anderson Cancer Center. Informed consent was obtained for the use of human samples.

### Animals

We purchased Balb/c (#000651), C57BL/6 (#000664), B6.129P2-Cxcr3^tm1Dgen^/J (005796), and Thy1.1 (#005443) mice from The Jackson Laboratory. FcƴRIIB^-/-^ mice (#Fcgr2b-Model 579) were purchased from Taconic. NOD.CB17-*Prkdc*^*scid*^/J (SCID) mice were a gift from Dr. Richard Gorlick (The University of Texas MD Anderson Cancer Center)^42^. Both female and male mice were used for all experiments. All mice were aged 5 to 8 weeks when the experimental procedures began. All animals were housed in a specific pathogen-free room with a 12-h light/dark cycle with free access to a standard rodent diet and water at ambient temperature maintained between 18 and 23 °C and humidity between 40 and 60%. We performed all animal experiments in accordance with the guidelines approved by the Institutional Animal Care and Use Committee (IACUC) at MD Anderson.

### Cells

DBT mouse glioma cells were kindly provided by Dr. Leonid Metelitsa (Baylor College of Medicine). GL261 cells were obtained from the National Cancer Institute. DBT-FGL2^−/−^ cells and GL261-FGL2^−/−^ cells were constructed as described previously^22^. 4T1 cells were purchased from ATCC. All cells were cultured as described previously^22,23^ and treated with a mycoplasma removal agent (BUF035, BIO-RAD) before experiments.

### Mouse models

For the orthotopic mouse models, DBT cells, GL261 cells, and 4T1 cells were treated with mycoplasma removal agent and collected in the logarithmic growth phase. Next, 5 × 10^4^ DBT cells, 5 × 10^4^ GL261 cells, or 5 × 10^4^ 4T1 cells in a total volume of 5 μL phosphate-buffered saline (PBS) were injected intracerebrally into Balb/c, C57BL/6, and Balb/c mice, respectively. For co-inoculations with T cells and DBT cells, 3 × 10^3^ DBT cells and 3 × 10^3^ T cells that had been sorted by flow cytometry were mixed and inoculated intracerebrally into naïve Balb/c mice. For co-inoculation of DBT cells with T_RM_-BILs, 3 × 10^3^ DBT cells and 3 × 10^4^ BILs cells that had been isolated as previously^22^ were inoculated intracerebrally into naïve SCID mice. The mice were observed daily. When the mice showed signs of neurological compromise, they were humanely euthanized by CO_2_ inhalation.

For the subcutaneous tumor model, 2 × 10^5^ DBT cells were suspended in 30 μL PBS and injected subcutaneously into 1 flank of each mouse. Tumors were measured twice per week. Mice that showed signs of morbidity, high tumor burden (diameter > 1 cm), or skin necrosis were immediately euthanized according to IACUC guidelines. Tumor volumes were calculated using the formula (length × width^2^)/2, with the length representing the longest axis, and the width at right angles to the length.

For in vivo blocking assays, the antibodies listed in Supplementary Table 2 were injected i.p. at the designated time to deplete or block the targets in vivo.

### Treatments

Starting on day 3 after tumor-cell inoculation, temozolomide (TMZ) from Accord Healthcare, Inc was injected intraperitoneally at a dose of 25 mg/kg (3 daily injections). On days 6 and 13 after tumor-cell inoculation, 2.5 million T cells transfected with either virus bearing a control vector or one bearing an FGL2-blocking scFv vector were injected intravenously.

### In vivo bioluminescence imaging

On a designated day after DBT tumor-cell implantation, mice were injected intraperitoneally with 150 mg/kg of D-luciferin in PBS. After 10 minutes, the mice were imaged with a charge-coupled device camera (IVIS 100; Xenogen-Caliper). Total photon flux (photons/s) was measured from a fixed region of interest over the skull using Living Image and IgorPro software (Wavemetrics).

### T cell preparation

Mouse T cells were isolated from the spleens and lymph nodes of Balb/c, C57BL/6, FGL2^−/−^, and LAG3^−/−^ mice and purified with a Mouse CD3 T cell Isolation Kit (480031; BioLegend). Human peripheral blood mononuclear cells (PBMCs) were isolated using Ficoll-Paque PLUS (GE Healthcare Biosciences AB) and SepMate-50 (Stemcell Technologies, Inc.) according to the manufacturers’ protocols. Human T cells were purified from PBMCs with a Human CD3 T Cell Isolation Kit (19051; BioLegend). Mouse T cells and human T cells were activated with Dynabeads Mouse T-Activator CD3/CD28 (0077118, Gibco) or Dynabeads Human T-Activator CD3/CD28 (00805147, Gibco) per the manufacturer’s instructions and then cultured in RPMI-1640 (Gibco), 10% fetal bovine serum (Hyclone), 2 mM GlutaMAX (Gibco), 100 μM β-mercaptoethanol (Gibco), 100 U/mL penicillin, and 100 μg/mL streptomycin (Corning) with 100 U interleukin (IL)−2 (Biolegend) and IL-15 (5 ng/mL) (Fisher). Twenty-four hours after activation, T cells were transfected with a lentivirus containing a control vector or an FGL2-blocking scFv vector. Cells were spun down at 1500 × *g* for 2 h with 8 μg/mL polybrene (MilliporeSigma) in RPMI medium in a retronectin (T100B; Takara)-coated 24-well plate. T cells were used for in vivo or in vitro experiments 2–4 days after transfection.

### In vitro cytotoxic T lymphocyte assay

For direct tumor cell killing assays, effector CD3^+^ T cells (2 days after activation) and target DBT-GFP^+^ tumor cells were cocultured at an E:T ratio of 4:1 for 72 h. For cytokine detection, target DBT tumor cells and effector CD3^+^ T cells (2 days after activation) were cocultured at an E:T ratio of 1:1 for 24 h. Four hours before cytokine detection, 10 μg/mL GolgiPlug (BD Biosciences) was added to the T cell medium.

### Flow cytometry

Mouse brain tissue and dLNs were minced and enzymatically digested to obtain single-cell suspensions. BILs were isolated per a previous protocol^43^. Briefly, each single-cell suspension was centrifuged through a 30% Percoll gradient at 7800 × *g* for 30 min. The leukocyte layer was collected and centrifuged on a discontinuous Ficoll-Paque Plus gradient to select and purify leukocytes. Leukocytes from mouse PB were collected as previously described^44^. Fc receptors were blocked using a rat anti-mouse anti-CD16/CD32 antibody (clone: 2.4G2, BD Biosciences). The antibodies used for flow cytometry are listed in Supplementary Table 3. The cell surfaces were stained by using a standard protocol. For intracellular cytokine staining, 4 h before the assay, Brefeldin A (1000× solution, Biolegend) was added at a 1:1000 dilution to the medium of in vitro cultured cells, and 250 μg Brefeldin A (AdipoGen Life Sciences) was injected into each mouse i.p for in vivo experiments. After cell surface staining, cells were fixed, permeabilized, and incubated with antibodies against IFNγ, TNFα, and granzyme B. For apoptosis assay, 7-AAD (Tonbo Biosciences, #13-6993-T500) and Annexin V antibody were used to stain cells in Annexin V binding buffer (BioLegend, #422201) for 15 min before detection. Stained cells were isolated by flow cytometry and the results analyzed by FlowJo software (version 10).

### FGL2 mAb screening

FGL2 mAbs were generated by the MD Anderson Monoclonal Antibody Core Facility. We performed ELISA to assay the binding reactivity of 75 clones of anti-FGL2 antibody and identify the clones that showed a high capacity to bind to recombinant mouse FGL2 protein. After that, 13 clones were left for subsequent screening. Next, diluted antibodies of these 13 clones were used for tests of their binding affinity to recombinant mouse FGL2 protein. Finally, the binding capacity to recombinant human FGL2 protein was measured to identify clones that reacted to both mouse and human FGL2.

### Plasmid construction and lentivirus production

Variable regions of a monoclonal antibody against FGL2 (clone #4) were sequenced by Genscript. FGL2 ScFV-mIgG2aFC-P2A-TM (transmembrane domain) was synthesized by Genscript and cloned into a third-generation self-inactivating lentiviral expression vector, pCDH (System Biosciences), with a cytomegalovirus promoter. The control virus was the same vector without the gene insert. High-titer replication-defective lentiviral vectors were produced and concentrated by the MD Anderson Functional Genomics Core and VectorBuilder, Inc..

### CyTOF

All mass cytometry reagents were purchased from Fluidigm, Inc. BILs were isolated from tumor-bearing brains and then washed with PBS containing 0.1% bovine serum albumin and blocked with a commercial Fc-blocking reagent (BD Biosciences) to minimize nonspecific antibody binding. The cells were then stained with a panel of metal-labeled antibodies (Supplementary Table 4) against cell surface markers for 30 minutes on ice and washed in PBS. After antibody staining, the cells were incubated for 5 min at room temperature (RT) with cisplatin as a viability dye for dead cell exclusion. The cells were then fixed and permeabilized with a FoxP3/Transcription Factor Staining Kit (eBioscience) according to the manufacturer’s protocol and stained with metal-labeled antibodies against intracellular cytokines and transcription factors. The cells were then washed and incubated overnight at 4 °C in PBS containing 1.6% formaldehyde and 125 nM Ir nucleic acid intercalator to label all nucleated cells. Immediately prior to acquisition, the cells were washed in diH_2_O, then resuspended in diH_2_O containing a 1/10 dilution of EQ 4 Element Calibration beads. The samples were acquired on a CyTOF2 mass cytometer. The resulting FCS files were concatenated and normalized using a bead-based normalization algorithm in the CyTOF acquisition software. FCS files were manually pre-gated on ^193^Ir DNA^+^CD45^+^ events, excluding cisplatin-positive dead cells, doublets, and DNA-negative debris.

The subsequent data processing and data transformation were done by using the flowCore^45^, by which the raw marker intensities were arcsinh-transformed (with cofactor 5) for cell clustering and further quantile-transformed (with 1st and 99th percentiles as the boundaries) for heatmap visualization. The clustering and cell population identification were conducted by using the FlowSOM^46^ and ConsensusClusterPlus^47^ packages for Bioconductor. The initially identified 15 cell clusters were visually represented by t-distributed stochastic neighbor embedding with a downsampling of 2000 cells per sample. Based on this, the delta area plot, and the heatmap of markers’ median expression levels for each cluster, we merged and annotated homogeneous clusters: neutrophils (CD11b^+^LY6G^+^), MDSCs (CD11b^+^Gr.1^+^), B cells (CD19 + ), MHCII^−^ macrophages (CD11b^+^F4/80^+^MHCII^−^), MHCII^+^ macrophages (CD11b^+^F4/80^+^MHCII^+^), monocytes (CD11b^+^LY6C^+^LY6G^−^), CD11b^+^ DCs (CD11c^+^MHCII^+^CD11b^+^), Ly6C^+^ DCs (CD11c^+^MHCII^+^Ly6C^+^), CD8^+^ proliferating T_EM_ cells (CD3^+^CD8^+^CD44^+^CD62L^−^Ki67^+^), CD8^+^ resting T_EM_ cells (CD3^+^CD8^+^CD44^+^CD62L^−^Ki67^−^), regulatory T cells (CD3^+^CD4^+^CD25^+^FoxP3^+^), helper T cells (CD3^+^CD4^+^CD25^−^FoxP3^−^), natural killer T cells (CD49b^+^CD3^+^), natural killer T cells (CD49b^+^CD3^−^), and CD4^−^CD8^−^ T cells (CD3^+^CD4^−^CD8^−^). The differential analysis of cell population abundance was conducted by fitting a generalized linear mixed model based on the binomial distribution, and a linear model was used for the differential analysis of marker expression.

### RNA sequencing

After 72-h coculture of T cells with DBT cells at a ratio of 4:1, T cells were collected and stained with anti-CD4 and anti-CD8 antibodies for 30 minutes at RT, then live CD8 + T cells were sorted with a FACSAria III flow cytometer (BD Biosciences). After spindown, cell pellets were frozen and shipped on dry ice to LC Sciences (Houston, TX) for total RNA extraction and total RNA next-generation sequencing (NGS) services. In general, total RNA was extracted using Trizol reagent (Thermo Fisher, 15596018) following the manufacturer’s procedure. After total RNA was extracted, mRNA was purified from total RNA (5 μg) using Dynabeads Oligo (dT) (Thermo Fisher) with two rounds of purification. Following purification, the mRNA was fragmented into short fragments using divalent cations under elevated temperature (94 °C) (Magnesium RNA Fragmentation Module; NEB, cat. e6150) for 5–7 min. Then the cleaved RNA fragments were reverse-transcribed to create the cDNA by SuperScript™ II Reverse Transcriptase (Invitrogen, cat. 1896649), which were next used to synthesize U-labeled second-stranded DNAs with *E. coli* DNA polymerase I (NEB, cat. m0209), RNase H (NEB, cat. m0297) and dUTP Solution (Thermo Fisher, cat. R0133). An A-base was then added to the blunt ends of each strand, preparing them for ligation to the indexed adapters. Each adapter contained a T-base overhang for ligating the adapter to the A-tailed fragmented DNA. Dual-index adapters were ligated to the fragments, and size selection was performed with AMPureXP beads. After treatment of the U-labeled second-stranded DNAs with the heat-labile UDG enzyme (NEB, cat. m0280), the ligated products were amplified with PCR. The average insert size for the final cDNA librarys were 300 ± 50 bp. Finally, paired-end sequencing (PE150) was performed on an Illumina Novaseq™ 6000 following the vendor’s recommended protocol.

### Alignment, quantification, and differential expression analysis of bulk sequencing data

Quality control was performed using fastQC (v. 0.11.9). Paired-end reads from fastq files were aligned and quantified with Salmon (v. 0.14.1)^48^ using gene annotation from GENCODE GRCm38. Salmon was used because it incorporates GC correction and accounts for fragment positional bias. After quantification, DESeq2 (v. 1.26.0) was used to perform differential expression analysis^49^.

### GSEA

Analysis of gene ontological features and signaling pathway enrichment was performed by using GSEA (v. 4.0.0)^50^. Enrichment terms with false discovery rate-adjusted *P* values less than 0.05 were considered statistically significant. Enrichment plots were plotted using GSEA.

### TCR sequencing

Lymphocytes from brain and dLN tissue were stained with anti-CD3, anti-CD44, and anti-CD8 antibodies for 30 minutes at RT and washed twice. Live CD3^+^CD44^+^CD8^+^ T cells were then sorted by using a FACSAria III flow cytometer (BD Biosciences) into 15-mL sterilized tubes containing 2 mL RPMI 1640 medium. After the samples were spun down, the supernatant was discarded. Next, the CD44^+^CD8^+^ T cells were resuspended in 1 mL RNAprotect Cell Reagent (Qiagen Inc.) and shipped on dry ice to iRepertoire, Inc. for RNA isolation and NGS of the TCRα and TCRβ immune repertoires. Sequences were analyzed by using the IMGT/V-QUEST web-based tool.

### Digital PCR assay

RNA was isolated from CD8^+^ T cells (sorted from T_RM_-bearing brains), T-Ctr cells (negative control of FGL2-scFv transfected T cells), and T-αFGL2 cells (positive control of FGL2-scFv transfected T cells) using Trizol reagent (Thermo Fisher Scientific). Total RNA was isolated from cells using an RNeasy Mini Kit (Qiagen). RNA was converted to cDNA using the IScript cDNA Synthesis Kit (BioRad). Droplet digital PCR (ddPCR) was performed using QX200™ ddPCR™ EvaGreen Supermix on the Bio-Rad QX200 system in the MD Anderson DNA Analysis Core Facility. Absolute quantification (ABS) was done using gene-specific primers and input cDNA from known cell numbers. Serial dilution of the #F4 containing plasmid was used as reference. The primer sequences for the #4 were: GCAACCTGGAGCAAGAAGAT; TCCACGGAGGAAGCGTATTA.

### Microfluidics slide chip assay

A PDMS microfluidics slide chip (Abnova) was coated with 1 mg/mL streptavidin (Agilent). The chip was then washed and coated with 4 mg/mL rmFGL2 (5257-FL, R&D Systems) conjugated with biotin for 1 h. Following this, 1.5 million T-Ctr cells (dyed green) and 1.5 million T-αFGL2 cells (dyed red) were loaded into a spiral chamber (Abnova) and passed through the slide chip at a flow rate of 1.8 mL per hour using the Cytoquest microfluidics pump (Abnova). The slide chip was then imaged on a fluorescent microscope (Keyence).

### T-αFGL2 toxicity studies in Balb/c immunocompetent mice

Six-week-old Balb/c mice were infused intravenously with syngeneic T cells transfected with control virus (T-Ctr) or FGL2-blocking scFv virus (T-αFGL2). Balb/c mice without T cell infusion were used as controls. Five days after T cell infusion, mice were euthanized, and their blood chemistry was assessed at VetMed LIMS. The immune cell composition of the spleen and bone marrow was assessed by flow cytometry. Tissue samples were also collected and subjected to hematoxylin and eosin (H&E) staining. A board-certified veterinary anatomic pathologist analyzed the stained slides at the Department of Veterinary Medicine and Surgery at MD Anderson.

### Western blotting

Cells were subjected to lysis in RIPA buffer (50 mM Tris-HCl, pH 7.4; 1% NP-40, 0.25% sodium deoxycholate, 150 mM NaCl, 1 mM EDTA) supplemented with 50 mM NaF, 20 mM β-glycerophosphate, and a complete protease inhibitor cocktail (Roche Diagnostics). Protein concentrations were determined using a bicinchoninic acid reagent (Thermo Fisher Scientific). Proteins were separated by sodium dodecyl sulfate-10% polyacrylamide gel electrophoresis and transferred to polyvinylidene fluoride membranes. Membranes were incubated overnight at 4 °C with primary antibodies. After washing, the blots were incubated with the appropriate horseradish peroxidase-conjugated secondary antibody and processed to detect electrochemiluminescence signals. Anti-FGL2 mAb-clone #4 was then applied for immunoblotting (1:500). The anti-FGL2 mAb-clone #4 was purchased from the MD Anderson Monoclonal Antibody Core Facility. α-β-Actin (13E5) and α-GAPDH (D16H11) antibodies were purchased from Cell Signaling Technology (1:1000). α-PIDD (Anto-1) antibodies were purchased from Novus Biologicals, Inc (1:500). For an example of full scan blots, see the Source Data file. The full information on the antibodies is provided in Supplementary Table 5.

### Immunohistochemistry

Mouse brains were fixed in formalin and embedded in paraffin, and 8-µm sections were cut for H&E staining. For anti-FGL2 antibody staining, mouse brain tissue samples were embedded in OCT compound and frozen in dry ice. After that, 8-µm sections were cut at −20 °C. Frozen sections of human tissue array sections, human GBM, and mouse brains were fixed with cold acetone, acetone/chloroform 1:1, and acetone for 5 min each, blocked with 3% H_2_O_2_ (Sigma) in distilled water for 20 minutes, then blocked with 1% BSA and 10% normal goat serum (Sigma) in PBS for 1 h at RT. Slides were stained with mouse anti-FGL2 antibody (1∶250, anti-FGL2 mAb-clone #4) overnight at 4 °C, followed by 1 h incubation with an anti-mouse biotin-conjugated antibody. Next, the sections were stained with a peroxidase-labeled anti-biotin antibody for 30 minutes and incubated with DAB for 5 minutes and Gill’s #3 hematoxylin for 30 s. Slides were dried in 75%, 95%, 100% ethanol and xylene for 2 minutes each and further processed for imaging analysis using a Keyence BZ-X800 microscope.

### Immunofluorescence

For immunofluorescence analyses of tumor cells, 2000 GL261 or GL261-FGL2^−/−^ cells were spun down onto slides separately, and then the slides were fixed with 4% paraformaldehyde for 10 minutes and permeabilized with 0.25% NP40 for 30 minutes at RT. After blocking with 5% horse serum and 1% goat serum in PBS for 1 h, slides were incubated with anti-FGL2 mAb-clone #4 at 4 °C overnight. The slides were washed 3 times with PBS, followed by 1 h incubation with anti-mouse Alexa Fluor 647-conjugated antibody. ProLong Gold Antifade Mountant with 4′-diamidino-2-phenylindole (DAPI; Thermo Fisher Scientific) was used as the mounting medium. A Leica SP8 confocal microscope was used for imaging analysis. For immunofluorescence analyses of frozen brain sections, after fixation and blocking as described in the Immunohistochemistry Staining section, 8-µm sections were stained with anti-CD3 antibody (1∶350, clone: SP7), anti-CD90.1 antibody (1:1000, clone:HIS51), anti-CD90.2 antibody(1:500, clone:53-2.1), and FITC-conjugated anti-His-tag antibody (1:1000, clone: 6-His) overnight at 4 °C. Following 3 washes with PBS, slides were incubated with goat anti-rabbit Alexa Fluor 647-conjugated antibody (1:1000, Thermo Fisher Scientific), goat anti-mouse Alexa Fluor 430-conjugated antibody (1:1000, Thermo Fisher Scientific), and goat anti-rat Alexa Fluor 568-conjugated antibody (1:1000, Thermo Fisher Scientific) for 1 h at room temperature. ProLong Gold Antifade Mountant with 4′-diamidino-2-phenylindole (DAPI; Thermo Fisher Scientific) was used as the mounting medium. A Leica SP8 confocal microscope was used for imaging analysis.

### Statistical analysis

Statistical significance was determined by unpaired Student *t*-test for pairwise comparisons or one-way ANOVA analysis of measurement data or Fisher’s exact test of enumeration data for comparing several treatments as indicated in the figure legends. For multiple unpaired *t* tests, the false discovery rate is shown along with the *p* value. Kaplan-Meier survival curves were analyzed using the log-rank test for multiple comparisons. *P*-value < 0.05 was considered statistically significant. Statistical analyses were performed using GraphPad Prism 8 software. Each figure legend indicates methods used for comparisons and correction.

### Reporting summary

Further information on research design is available in the Nature Portfolio Reporting Summary linked to this article.

## Supplementary information

Supplementary Information

## Data Availability

The raw TCR-seq data are available in the GEO database under the accession number GSE218756 and the raw bulk RNA-seq data are available in the GEO database under the accession number GSE218639. Source data are provided in this paper. The raw numbers for charts and graphs are available in the Source Data file whenever possible. The authors declare that all data supporting the findings of this study are available within the paper and its Supplementary Information files or from the corresponding author upon reasonable request. Source data are provided as a Source Data file. Source data are provided with this paper.

## Source data

Source Data
Reporting Summary
