# Supplementary material for: FGL2-targeting T cells exhibit antitumor effects on glioblastoma and recruit tumor-specific brain-resident memory T cells
Source: Nat Commun. 2023 Feb 10;14:735. doi: 10.1038/s41467-023-36430-2 (PMC9911733; doi:10.1038/s41467-023-36430-2)
Supplement: Supplementary file 1 — Supplementary Information [file 41467_2023_36430_MOESM1_ESM.pdf]

FGL2-targeting T cells exhibit anti-tumor effects on glioblastoma and recruit tumor-specific brain-resident memory T cells

Qingnan Zhao[1,2,3], Jiemiao Hu[2], Lingyuan Kong[2], Shan Jiang[4], Xiangjun Tian[5], Jing Wang[5], Rintaro Hashizume[6], Zhiliang Jia[2], Natalie Wall Fowlkes[7], Jun Yan[8], Xueqing Xia[2], Sofia F Yi[2], Long Hoang Dao[2], David Masopust[9], Amy B. Heimberger[5], and Shulin Li[2#]

[1]*Department of Clinical Pharmacy, Shanghai General Hospital, Shanghai Jiao Tong University School of Medicine, Shanghai, 200020, China*

[2]*Division of Pediatrics, The University of Texas MD Anderson Cancer Center, Houston, TX 77030 USA*

[3]*Shanghai Key Laboratory of Pancreatic Disease, Shanghai Jiao Tong University School of Medicine, Shanghai, 201620, China*

[4]*Center for Precision Health, School of Biomedical Informatics, The University of Texas Health Science Center at Houston, Houston, TX 77030, USA.*

[5]*Department of Bioinformatics and Computational Biology, The University of Texas MD Anderson Cancer Center, Houston, TX, 77030, USA*

[6]*Department of Neurological Surgery, Malnati Brain Tumor Institute of the Lurie Comprehensive Cancer Center, Feinberg School of Medicine, Northwestern University, Chicago, IL 60611, USA.*

[7]*Department of Veterinary Medicine & Surgery, The University of Texas MD Anderson Cancer Center, Houston, TX 77030 USA*

[8]*Center for Brain Disorders Research, Capital Medical University, Beijing, 100069, China*

[9]*Department of Microbiology, Center for Immunology, University of Minnesota, Minneapolis, MN 55455, USA*

[#]**Corresponding author,** *Shulin Li, PhD, Professor, Department of Pediatrics–Research, Unit 853, The University of Texas MD Anderson Cancer Center, 1515 Holcombe Boulevard, Houston, TX 77030, USA, E-mail: Sli4@mdanderson.org; Tel.: 713-563-9608, fax: 713-563-9607*

Supplementary data

Supplementary Fig. 1

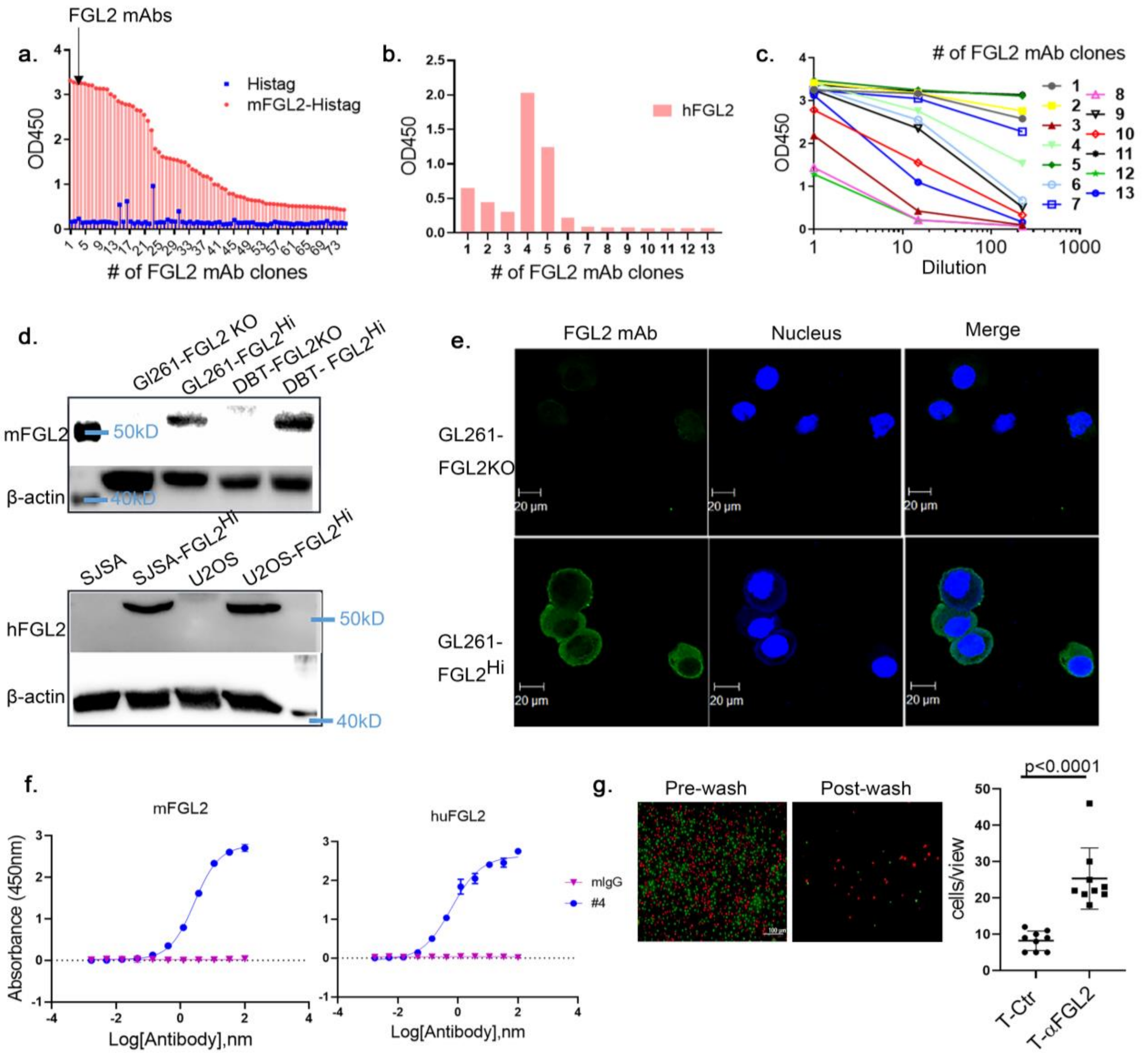

Supplementary Fig. 1. Screening of anti-FGL2 monoclonal antibodies. a, Initial screen of the binding capability of anti-FGL2 monoclonal antibodies (mAbs) to recombinant mFGL2 protein with a His tag or His tag alone via ELISA. **b**, Screening of the top 13 anti-FGL2 mAb clones from **a**. Binding affinity of the diluted mAbs to recombinant mFGL2 protein with a His tag was determined using ELISA. **c**, Screening of binding capability of anti-FGL2 mAbs to recombinant hFGL2 protein. Clone #4 bound to both mouse and human FGL2. Western blotting (**d**) and immunofluorescence staining (**e**) were used to validate the binding reactivity of anti-FGL2 mAb-clone #4 to mFGL2. Data are representative of three independent experiments. **f**, ELISA was used to confirm the binding affinity of clone #4 to hFGL2 and mFGL2 (n=3), data shown are mean ± SD. **g**, 1.5 million T-Ctr cells (dyed green) and 1.5 million T-αFGL2 cells (dyed red) were loaded into a FGL2 coated chamber and passed through the slide

chip using the Cytoquest microfluidics pump. The slide chip was then imaged on a fluorescent microscope (n=9/group, data represents mean ± SD), two-way t-test. Data are representative of two independent experiments.

Supplementary Fig. 2

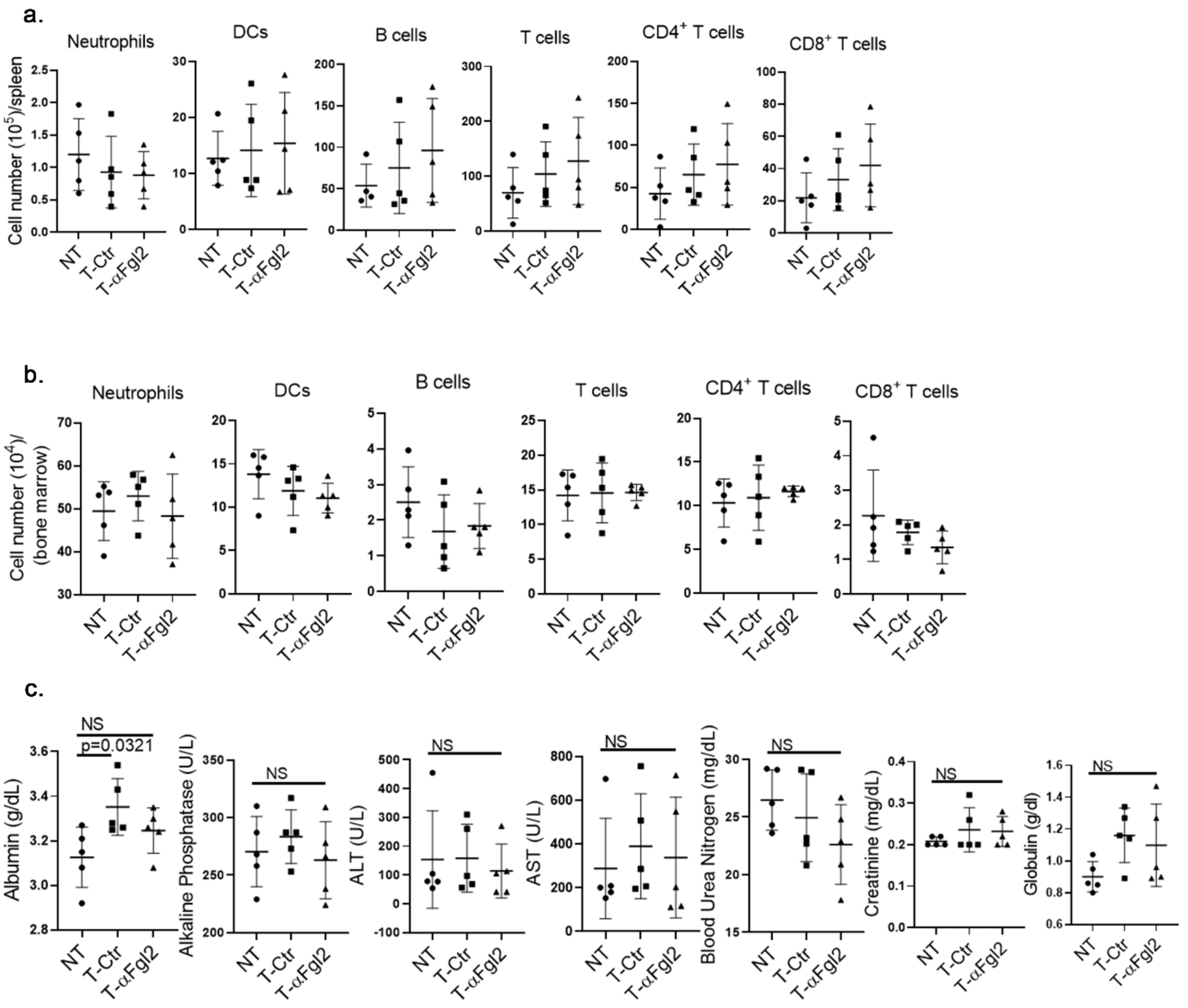

Supplementary Fig. 2. T-αFGL2 therapy does not cause toxicity *in vivo*. Balb/c mice were infused intravenously with 5×10^6 T-αFGL2 cells or T-Ctr cells, or no treatment (NT). Five days later, mice were euthanized for flow cytometry analysis of the immune cell composition of the spleen (a) and bone marrow (b). Blood was collected to assess blood chemistry (c). Experiment was performed once with n=5, data represent mean ± SD. NS, not significant, one-way ANOVA with Turkey's test for comparing multiple treatments. DCs, dendritic cells, ALT, alanine transaminase; AST, aspartate aminotransferase.

Supplementary Fig. 3

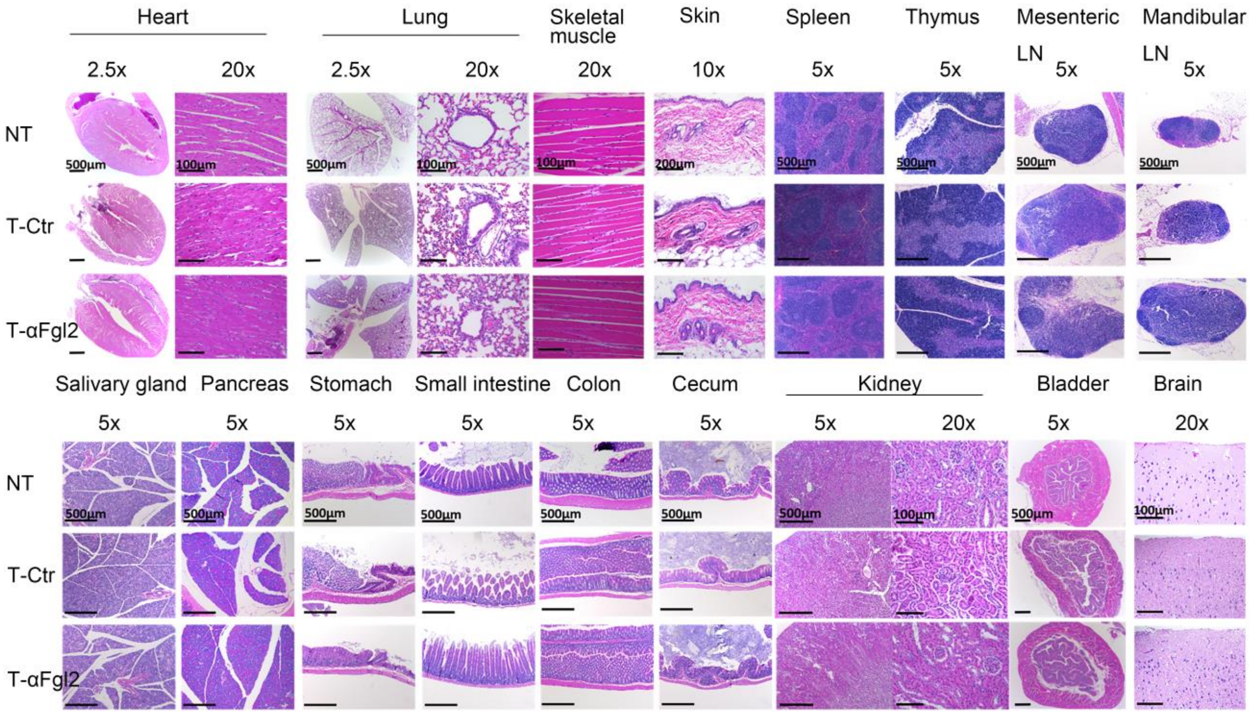

Supplementary Fig. 3. T-αFGL2 does not cause obvious abnormal changes in tissues. Representative micrographs showing hematoxylin and eosin staining of the indicated tissues collected from Balb/c mice 5 days after intravenous infusion with 5×10^6 T-Ctr or T-αFGL2 cells. LN, lymph nodes; NT, no treatment. Data are representative of different group of Balb/c mice were infused intravenously with 5×10^6 T-αFGL2 cells or T-Ctr cells, or no treatment (NT) (Experiment was performed once with n=5 mice/group).

Supplementary Fig. 4

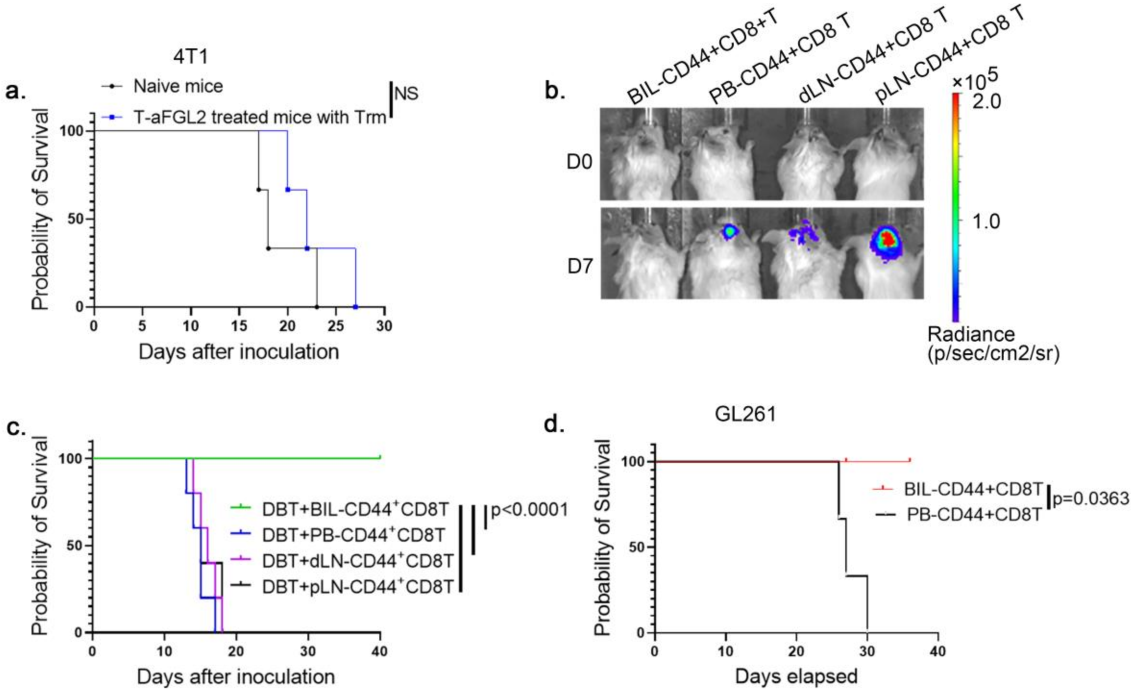

Supplementary Fig. 4. T-αFGL2 treatment induced TRM cells are transplantable and tumor specific. a, Kaplan-Meier survival curves for DBT tumor–rejecting mice inoculated (i.c.) with 1×10^4 4T1 cells (*n* = 3 mice/group). **b**, Representative bioluminescence images of naïve mice co-inoculated i.c. with 3×10^3 DBT glioma cells and 3×10^3 CD44+ memory CD8T cells. Images show gliomas in mice co-inoculated with CD44+ memory CD8 T cells in the brain (BIL-CD44+CD8T), PB (PB-CD44+CD8T), dLNs (dLN-CD44+CD8T), and pLNs (pLN-CD44+CD8T). CD8+ T cells and CD4+ T cells were sorted by flow cytometry on day 7 after the third challenge in T-αFGL2 survivors. **c**, Kaplan-Meier survival curves of mice in d (n = 5 mice/group), log-rank test. dLNs: draining LNs (cervical LNs); pLNs: pooled peripheral LNs (mediastinal LNs, axillary LNs, brachial LNs, and inguinal LNs); PB: peripheral blood. **d**, Kaplan-Meier survival curves for C57 mice co-inoculated with 1×10^3 GL261 and 3×10^3 CD44+CD8+ T cells from the brain (BIL-CD44+CD8+T) and peripheral blood (PB-CD44+CD8+T). Cells were sorted by flow cytometry on day 6 after the second GL261 challenge in T-αFGL2 survivors, NS. not significant, log-rank test.

Supplementary Fig. 5

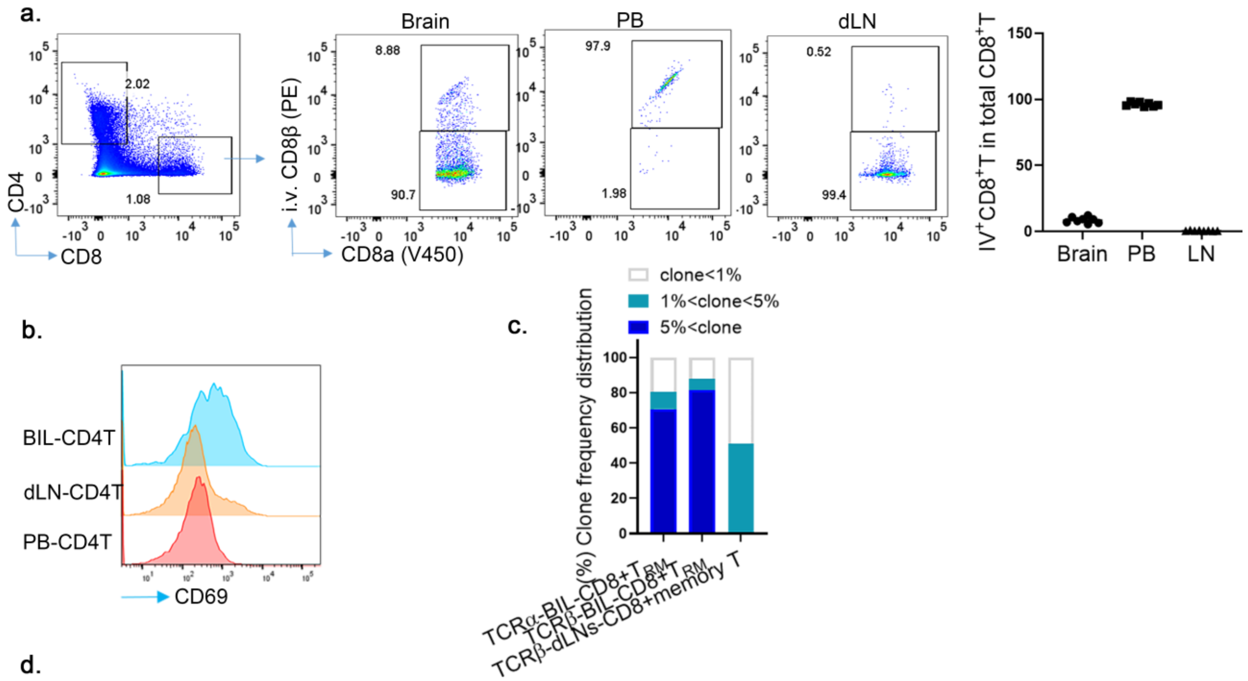

d.

TCRβ-BIL-CD44+CD8+T		TCRβ-dLNs-CD44+CD8+T	
CDR3 sequence	Frequency (%)	CDR3 sequence	Frequency (%)
ASSRYRTGEGERLF	97.55983	AWRTNNSPLY	4.308103747
ASSRYRAGEGERLF	0.500547	AWSLWAETLY	3.230144027
ASSRYRTGGGERLF	0.344126	AWKDWGGALSYEQY	2.628787426
ASSRYRTGEGGRLF	0.344126	ASSETGRVF	2.398329648
ASSRYGTGEGERLF	0.328484	ASGGDISGNTLY	2.248550768
ASSRCRTGEGERLF	0.312842	ASSDAGGSAETLY	2.140978904
ASSRHRTGEGERLF	0.234632	ASSLGPYEQY	2.116327018
ASSGYRTGEGERLF	0.109495	ASSSGTSNTEVF	2.084951891
ASSRYRTGEGARLF	0.046926	ASSDQTGVTEVF	2.012863802
ASSRYKTGESERLF	0.046926	ASSLLGGNNYAEQF	1.998670292

Supplementary Fig. 5. BIL-T cells were mainly brain resident T cells. a, Intravital (IV) staining of $CD8^{+}$T cells showing $CD8^{+}$ T cells from brain, blood, and dLNs ($n = 8$, representing three experiments, data represents mean ± SD). **b**, Representative flow cytometry plots and graphs showing CD69 expression on $CD4^{+}$ T cells in the brain, dLN, and PB of T-αFgl2–treated survivors (DBT model). **c**, Clone frequency distributions of TCRα-BIL-$CD8^{+}T_{RM}$, TCRβ-BIL-$CD8^{+}T_{RM}$, TCRβ-dLNs-$CD44^{+}CD8^{+}$T clones based on CDR3 sequences of DBT model. Highly expanded TCRα/β clones (frequency > 5%) constituted more than 60% of BIL-$CD8^{+}T_{RM}$ population. d, Top 10 CDR3 sequences of TCRβ-BIL-CD44+CD8+T and TCRβ-dLNs-$CD44^{+}CD8^{+}$T of DBT model.

Supplementary Fig.6

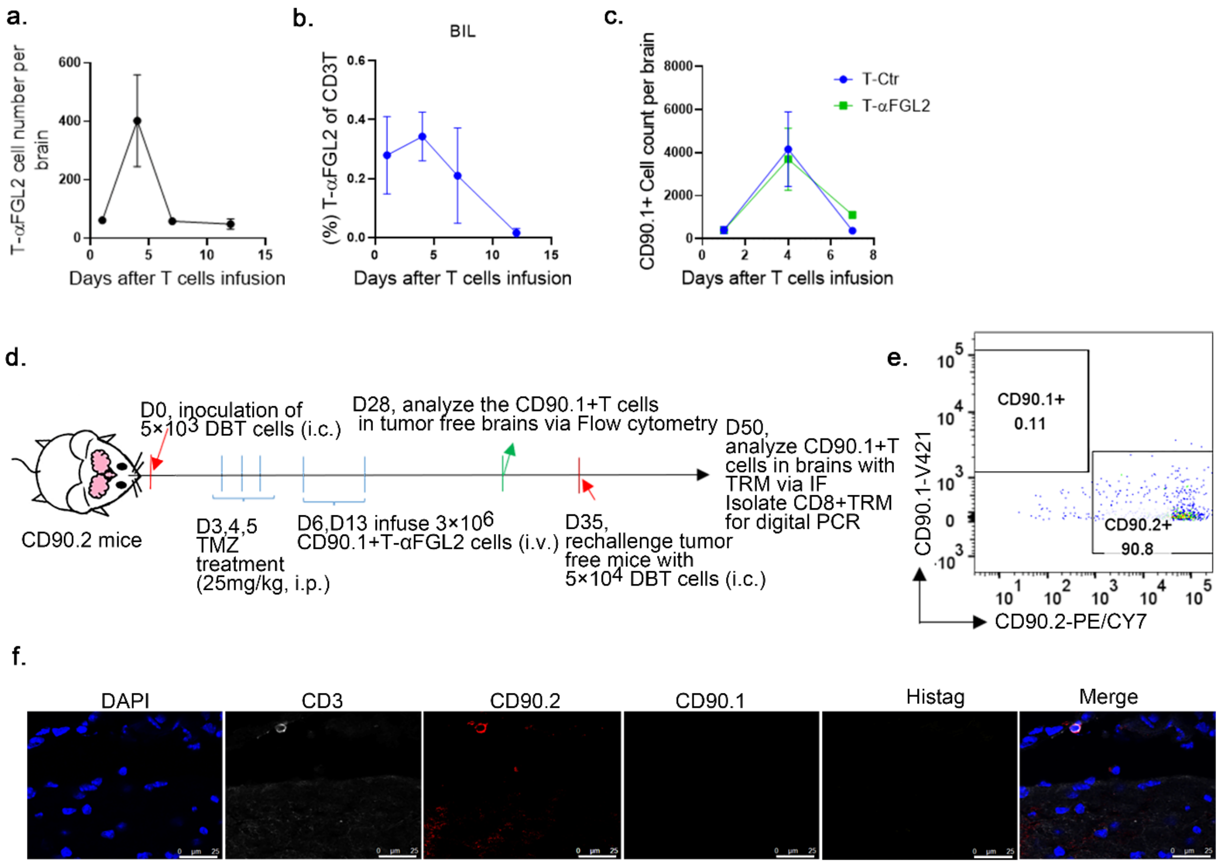

g.

Sample	RNA input	cell # in RXN	plasmid molecules	Positives	Negatives	total Droplets
T-Ctr	4ng	1600		0	10354	10354
T-αFGL2	4ng	2240		2524	7182	9706
**CD8+TRM**	**1.2ng**	**1000**		**0**	**9408**	**9408**
αFGL2 plasmid			3	0	9896	9896
αFGL2 plasmid			30	0	11868	11868
αFGL2 plasmid			300	427	8166	8593
αFGL2 plasmid			3000	4270	4574	8844

Supplementary Fig. 6. T-αFGL2 infiltrated into tumor bearing brains but did not persist for long. A total of 3×10^6 CD90.1$^+$T cells transduced with lentivirus encoding control or αFGL2-scFv-His tag (T-Ctr or T-αFGL2) were injected (i.v.) into DBT bearing CD90.2 mice. CD90.1$^+$T cells were detected on days 1, 4, 7, 12 after injection. The number (**a**) of CD90.1$^+$T-αFGL2 cells and the percentage of these cells among total CD3$^+$T cells in tumor bearing brains (**b**) were detected on day 1 after T cell injection (n=3 on day 1, 4, 12; n=2 on day 7). Data represents mean ± SEM. The population peaked on day 4 and declined at day 7 and day 12. The number of CD90.1$^+$T cells (**c**) in tumor

bearing brains increased from day 1 to day 4, and then decreased from day 4 to day 7. Data represents mean ± SEM). **d**, Flow chart of experiment; **e**, Flow cytometry data of $CD90.1^+$ and $CD90.2^+$ T cells in tumor free brains on day 15 after the last $CD90.1^+$T-αFGL2 treatment; **f**, IF staining of T_{RM} bearing brains with αCD3, αCD90.1, αCD90.2, and αHis tag Abs on day 15 after DBT rechallenge, data are representative of 2 independent experiments, scale bars=25μm; **g**, Digital PCR analysis of αFGL2-scFv sequences in CD8+T_{RM} cells isolated from tumor free brains on day 15 after DBT rechallenge. T-Ctr and T-αFGL2 samples were used as negative and positive controls, respectively, and a different amount of αFGL2 plasmid was used as a standard.

Supplementary Fig. 7

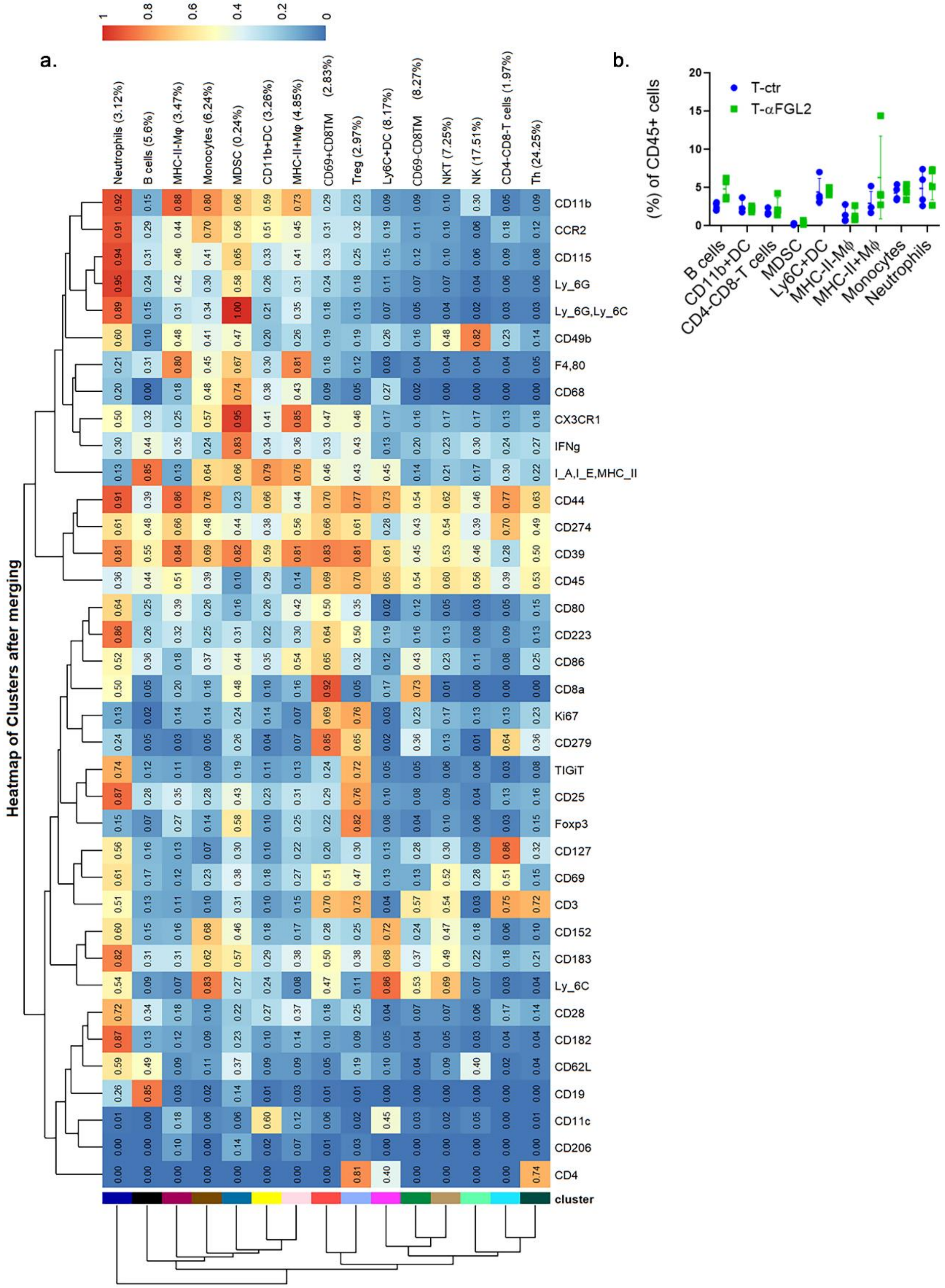

Supplementary Fig.7. Immune cell clusters and cluster frequency in brain of CyTOF data. **a**,Heatmap of different markers' expression on 15 clusters of CD45^{+} cells in brain of CyTOF data in Fig. 7; **b**, Frequency of different cell populations in brains with T-Ctr or T-αFGL2 treatment ($n = 4$, data represent mean ± SD).

Supplementary Fig. 8

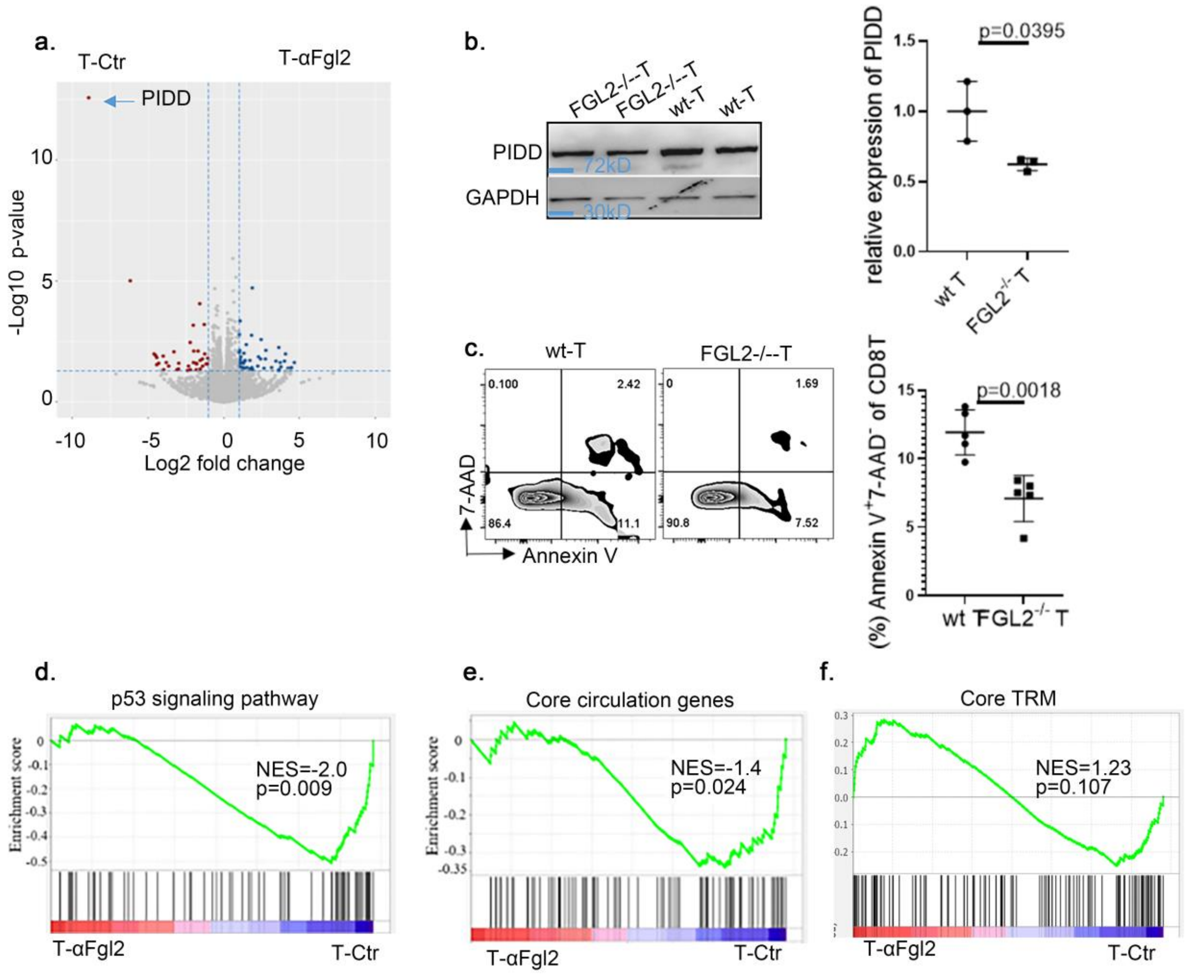

Supplementary Fig. 8. T-αFGL2 diminished gene transcripts associated with apoptosis and circulation.
a, Volcano plot of RNA next-generation sequencing analysis of differentially expressed genes in CD8$^+$ T-αFGL2 and CD8$^+$ T-Ctr cells cocultured with DBT cells at an effector-to-target ratio of 4:1 for 72 h. Western blots of PIDD expression **(b,** n=3, data represents mean ± SD**)** and Annexin V staining **(c,** n=5, data represents mean ± SD**)** of wild-type T cells and FGL2$^{-/-}$ T cells activated with α-CD3/CD28 for 4 days; p values from two-way *t*-test. GSEA of genes involved in the P53 signaling pathway (**d**), core circulation genes, and core T$_{RM}$ genes (**e**) in CD8$^+$ T-αFGL2 and CD8$^+$ T-Ctr cells.

Supplementary Fig. 9

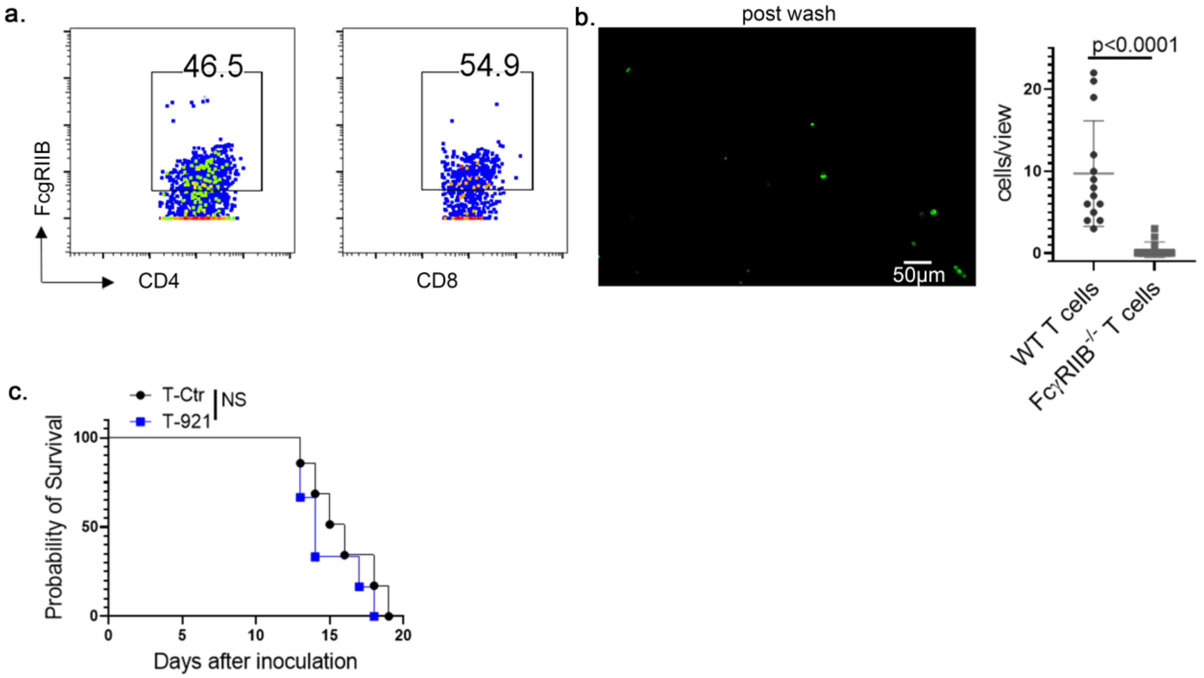

Supplementary Fig. 9. FcγRIIb expressed on the T cells and responded to FGL2

a, Representative CYTOF data of FcγRIIb expression on $CD4^{+}$T cells and $CD8^{+}$T cells in tumor bearing brains (n=6/group). **b**, 1.5 million WT T cells (dyed green) and 1.5 million FcγRIIb$^{-/-}$ T cells (dyed red) were loaded into a FGL2 coated chamber and passed through the slide chip using the Cytoquest microfluidics pump. The slide chip was then imaged on a fluorescent microscope (n=14, data represents mean ± SD), two-way t-test. Data are representative of 2 independent experiments. **c**, Kaplan-Meier survival curves for DBT bearing FcγRIIb$^{-/-}$ mice treated with T-Ctr or T-αFGL2, NS not significant, log-rank test.

Supplementary Fig. 10

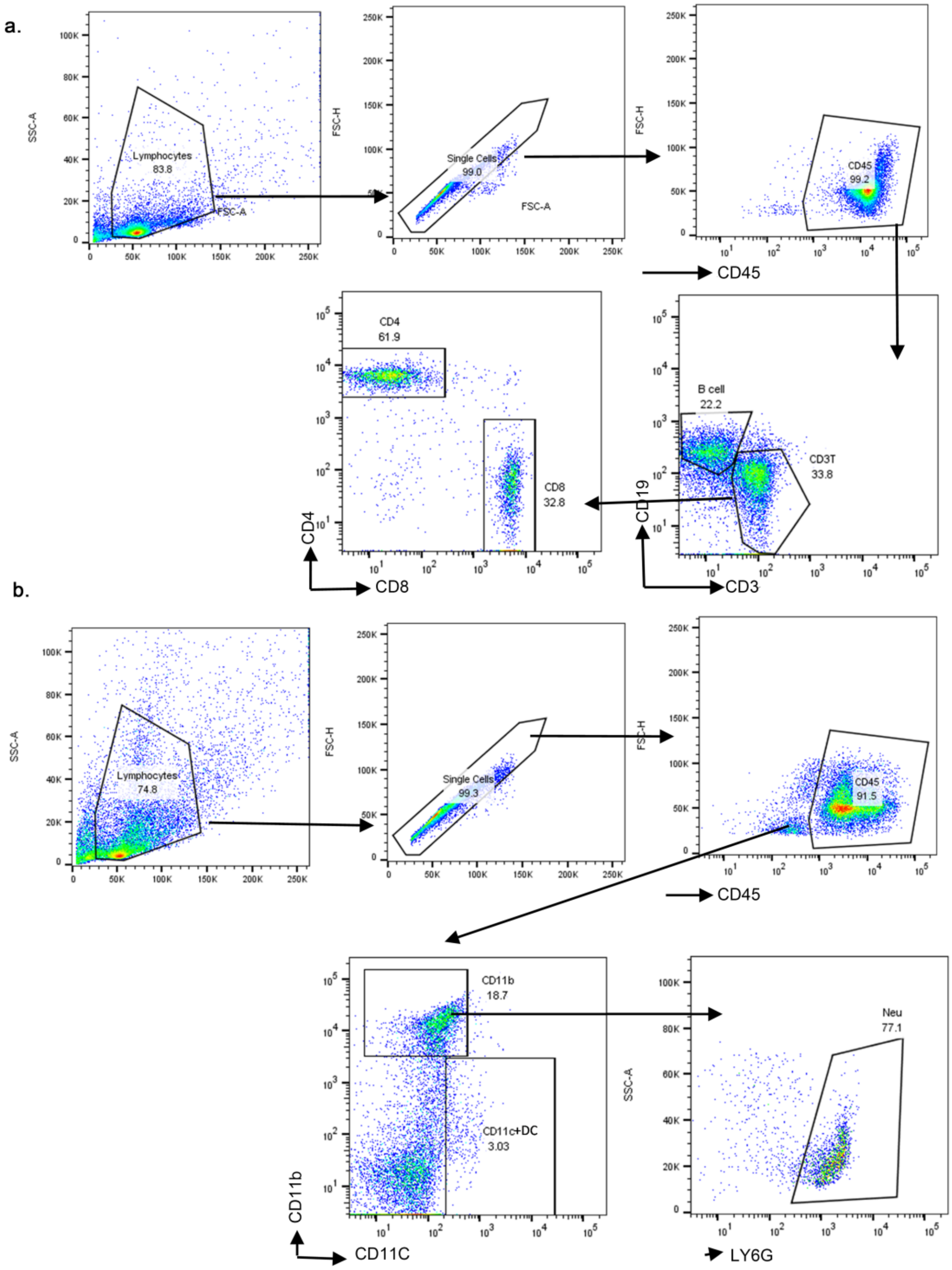

Supplementary Fig. 10. Flow cytometry gating strategy of different cell populations. a, Gating strategy for B cells, T cells, CD8⁺T cells, and CD4⁺T cells; **b**, Gating strategy for dendritic cells (DCs) and neutrophils.

Supplementary Table 1. Histopathologic characteristics and diagnoses for mice treated with T-Ctr, T-αFGL2, or no treatment (NT).

Organ Morphologic Diagnosis	NT	T-Ctr	T-αFGL2	P value
**Liver**	N (100%)	N (100%)	N (100%)	1
**Increased mitoses**	P (20%)	P (0%)	P (0%)	1
**Mononuclear infiltrate portal area, focal**	1 (60%)	1 (20%)	1 (20%)	0.498
**Kidney**	N (100%)	N (100%)	N (100%)	1
**Lung**	N (100%)	A (40%)	N (100%)	0.285
**Mononuclear infiltrate**	0 (100%)	1 (20%)	0 (100%)	1
**Inflammation, vessel wall**	0 (100%)	P (20%)	0 (100%)	1
**Heart**	N (100%)	A (20%)	A (20%)	1
**Inflammation, vessel wall**	0 (100%)	P (20%)	0 (100%)	1
**Inflammation, myocardium**	0 (100%)	1 (20%)	1 (20%)	1
**Brain**	N (100%)	N (100%)	N (100%)	1
**Skeletal muscle**	N (100%)	N (100%)	N (100%)	1
**Duodenum, Jejunum, lleum**	N (100%)	N (100%)	N (100%)	1
**Mesenteric Lymph Node and Mesentery**	N (100%)	N (100%)	N (100%)	1
**Spleen**	N (100%)	N (100%)	N (100%)	1
**Salivary Gland**	N (100%)	N (100%)	N (100%)	1
**Trachea**	N (100%)	N (100%)	N (100%)	1
**Esophagus**	N (100%)	N (100%)	N (100%)	1
**Skin**	N (100%)	N (100%)	N (100%)	1
**Colon**	N (100%)	N (100%)	N (100%)	1
**Stomach**	N (100%)	N (100%)	N (100%)	1
**Pancreas**	N (100%)	N (100%)	N (100%)	1

Legend: N= Normal tissue or No significant lesion observed; A= Abnormal tissue (Lesion present); P= The lesion is present, but not graded. Grading/Scoring of histological lesions; Grade 0=no histologic lesion (or normal tissue); Grade1=minimal, minor, rare, infrequent, barely noticeable tissue change (lesion affects 1-10% of the tissue). p values from Fisher's exact test (n=5).

Supplementary Table 2. Antibodies used for *in vivo* blocking.

Target	Source	Cat #	Dilutions
Rat IgG (clone: 2A3)	BioXcell	BE0089	100~200µg/mice
CD8 (clone: 2.43)	BioXcell	BE0061	100µg/mice
CD4 (clone: GK1.5)	Leinco Techonologies	25-0032-U100	100µg/mice
Asialo GM1	Fujifilm wako chemical U.S.A. corporation	986-10001	15µl/mice
CD69 (clone: H1.2F3)	Thermo Fisher scientific	14-0691-82	150µg/mice
MHC-I (clone: M1/42.3.9.8)	BioXcell	BE0077	200µg/mice
CXCL9 (clone: MIG-2F5.5)	BioXcell	BE0309	200µg/mice
CXCL10 (clone: 134013)	Thermo Fisher scientific	MA5-23774	200µg/mice

Supplementary Table 3. Antibodies used for flow cytometry analysis.

Target	Source	Cat #	Dilutions
CD4-PE-CY7 (clone: RM4-5)	Tonbo Biosciences	60-0042-U100	0.5µg/100µl
CD8α-VF450 (clone: 53-6.7)	Tonbo Biosciences	75-0081-U100	0.5µg/100µl
CD3-APC/CY7 (clone: 17A2)	Tonbo Biosciences	25-0032-U100	0.5µg/100µl
CD8α-APC/CY7 (clone: 53-6.7)	Tonbo Biosciences	25-0081-U100	0.5µg/100µl
CD8α-FITC (clone: 53-6.7)	BioLegend	100706	0.5µg/100µl
CD8α-PE (clone: 53-6.7)	Tonbo Biosciences	50-0081-U100	0.5µg/100µl
CD8β-PE (clone: YTS156.7.7)	BioLegend	126607	0.5µg/100µl
CD11b-PE (clone: M1/70)	Tonbo Biosciences	50-0112-U100	0.5µg/100µl
CD11c-FITC (clone: N418)	BioLegend	117305	0.5µg/100µl
Ly6G-APC/CY7 (clone: 1A8)	BioLegend	127623	0.5µg/100µl
CD19-FITC (clone: 1D3)	Tonbo Biosciences	35-0193-U025	0.5µg/100µl
CD45-Pacific blue (clone: 30-F11)	BioLegend	103125	0.5µg/100µl
TNFα-PE (clone: MP6-XT22)	BioLegend	506306	1µg/100µl
Granzyme B-PE (clone: QA16A02)	BioLegend	372208	1µg/100µl
IFNγ-VF450 (clone: XMG1.2)	Tonbo Biosciences	75-7311-U100	1µg/100µl
CD69-FITC (clone: H1.2F3)	BioLegend	104506	0.5µg/100µl
CD103-PE (clone: 2E7)	BioLegend	121406	0.5µg/100µl
CD44-FITC (clone: 1M7)	BioLegend	103021	0.5µg/100µl
CD62L-APC (clone: MEL-14)	BioLegend	104412	0.5µg/100µl
CD183-PE (clone: CXCR3-173)	BioLegend	126511	0.5µg/100µl
CD183-APC (clone: CXCR3-173)	BioLegend	126505	0.5µg/100µl
B220-PE/CY7 (clone:RA3-6B2)	BioLegend	103221	0.5µg/100µl
CD90.1-PE (clone:HIS51)	Thermo Fisher scienctific	12-0900-81	0.5µg/100µl
CD90.2-eFluor™ 450 (clone:53-2.1)	Thermo Fisher scienctific	48-0902-82	0.5µg/100µl

Annexin V-Pacific blue	BioLegend	640918	5µl/100µl
Fixable Viability Dye eFluor™ 780	Thermo Fisher scienctific	65-0865-14	1µl/500µl

Supplementary Table 4. Antibodies used for CyTOF analysis *in vivo*.

Target	Label	Clone	Specificities	Source	Cat #	Dilutions
CD45 (Ms)	^{89}Y	30-F11	Ms	DVS-Fluidigm	3089005B	1µl/100µl
CD4 (Ms)	^{115}In	RM4-5	Ms	BioLegend	100506	1µl/100µl
Ly-6G/Ly-6C, Gr-1	^{139}La	RB6-8C5	Ms	BioLegend	108402	1µl/100µl
Ly-6G	^{141}Pr	1A8	Ms	DVS-Fluidigm	3141008B	1µl/100µl
CD39	^{142}Nd	24DMS1	Ms	DVS-Fluidigm	3142005B	1µl/100µl
CCR2	^{143}Nd	475301	Ms	R&D Systems	MAB55381	1µl/100µl
CD115	^{144}Nd	AFS98	Ms	DVS-Fluidigm	3144012B	1µl/100µl
CD68	^{145}Nd	FA-11	Ms	BioLegend	137002	1µl/100µl
CD8a (Ms)	^{146}Nd	53-6.7	Ms	DVS-Fluidigm	3146003B	1µl/100µl
CD223, LAG-3	^{147}Sm	C9B7W	Ms	BioLegend	125202	1µl/100µl
CD11b (Ms)	^{148}Nd	M1/70	Ms	DVS-Fluidigm	3148003B	1µl/100µl
CD19 (Ms)	^{149}Sm	6D5	Ms	DVS-Fluidigm	3149002B	1µl/100µl
CD25	^{150}Nd	3C7	Ms	BioLegend	101902	1µl/100µl
TIGiT, Vstm3	^{151}Eu	1G9	Ms	BioLegend	142102	1µl/100µl
CD3, CD3e	^{152}Sm	145-2C11	Ms	BioLegend	100302	1µl/100µl
CD182, CXCR2	^{153}Eu	SA044G4	Ms	BioLegend	149302	1µl/100µl
CD274, PD-L1	^{154}Sm	10F.9G2	Ms	BioLegend	124303	1µl/100µl
CD69	^{156}Gd	H1.2F3	Ms	BioLegend	104533	1µl/100µl
Foxp3	^{158}Gd	FJK-16s	Ms, Rt, Bv, Cn, Po, Fe	DVS-Fluidigm	3158003A	1µl/50µl
CD279, PD-1	^{159}Tb	29F.1A12	Ms	BioLegend	135202	1µl/100µl
CD62L	^{160}Gd	MEL-14	Ms	DVS-Fluidigm	3160008B	1µl/100µl
CD183, CXCR3	^{161}Dy	CXCR3-173	Ms	BioLegend	126502	1µl/100µl

Ly-6C	^{162}Dy	HK1.4	Ms	DVS-Fluidigm	3162014B	1µl/100µl
CD152, CTLA-4	^{163}Dy	9H10	Ms	BioLegend	106202	1µl/100µl
CD49b	^{164}Dy	HMa2	Ms	DVS-Fluidigm	3164011B	1µl/100µl
IFNg	^{165}Ho	XMG1.2	Ms	DVS-Fluidigm	3165003B	1µl/100µl
CD44	^{166}Er	IM7	Hu, Ms, Ch, Rh	BioLegend	103002	1µl/100µl
Ki67	^{168}Er	B56	Ms, Hu	BD	556003	1µl/50µl
CD206, MMR	^{169}Tm	C068C2	Ms	DVS-Fluidigm	3169021B	1µl/100µl
CD127, IL-7Ra	^{170}Er	A7R34	Ms	BioLegend	135029	1µl/100µl
CD80	^{171}Yb	16-10A10	Ms, Cn	DVS-Fluidigm	3171008B	1µl/100µl
CD86	^{172}Yb	GL1	Ms	DVS-Fluidigm	3172016B	1µl/100µl
F4/80	^{173}Yb	BM8	Ms	BioLegend	123102	1µl/100µl
I-A/I-E MHC-II	^{174}Yb	M5/114.15.2	Ms	DVS-Fluidigm	3174003B	1µl/100µl
CD28	^{175}Lu	37.51	Ms	BioLegend	102119	1µl/100µl
CX3CR1	^{176}Yb	SA011F11	Ms	BioLegend	149002	1µl/100µl
CD11c	^{209}Bi	N418	Ms	DVS-Fluidigm	3209005B	1µl/100µl

Abbreviations: Ms, mouse; Rt, rat; Bv, bovine; Cn, canine; Po, porcine; Fe, feline; Hu, human; Ch, Chicken; Rh, rhesus

Supplementary Table 5. Antibodies used for Western blotting, Immunohistochemistry and Immunofluorescence

Target	Clone	Specificities	Source	Cat #	Dilutions
FGL2	#4	Ms/Hu	MD Anderson Monoclonal Antibody Core Facility		1:500 for WB<br>1:250 for IHC, IF
β-Actin	13E5	Hu/Ms/Rt/Mk	Cell Signalling Technology	5057	1:1000
GAPDH	D16H11	Hu/Ms/Rt/Mk	Cell Signalling Technology	5174	1:1000
PIDD	Anto-1	Ms/Hu	Novus Biologicals, Inc	NBP1-97595	1:500
CD3	SP7	Ms/Hu/Rt	Thermo Fisher scienctific	MA1-90582	1:350
CD90.1	HIS51	Ms/Rt	Thermo Fisher scienctific	14-0900-81	1:1000
CD90.2	53-2.1	Ms	Thermo Fisher scienctific	14-0902-82	1:500
Histag	J095G46	Ms/Hu/Rt	BioLegend	362618	1:1000
IgG		Rab	Thermo Fisher scienctific	A-21245	1:1000
IgG		Ms	Thermo Fisher scienctific	A-11063	1:1000
IgG		Rt	Thermo Fisher scienctific	A-11077	1:1000

Abbreviations: Ms, mouse; Rt, rat; Hu, human; MK, monkey; Rab, Rabbit; WB, western blotting; IHC, immunohistochemistry; IF, immunofluorescence.
