# Supplementary material for: FGL2-targeting T cells exhibit antitumor effects on glioblastoma and recruit tumor-specific brain-resident memory T cells
Source: Nat Commun. 2023 Feb 10;14:735. doi: 10.1038/s41467-023-36430-2 (PMC9911733; doi:10.1038/s41467-023-36430-2)
Supplement: Supplementary file 2 — Source Data [file 41467_2023_36430_MOESM2_ESM.pdf]

nature portfolio

Corresponding author(s): Shulin Li

Last updated by author(s): Nov 28, 2022

Reporting Summary

Nature Portfolio wishes to improve the reproducibility of the work that we publish. This form provides structure for consistency and transparency in reporting. For further information on Nature Portfolio policies, see our Editorial Policies and the Editorial Policy Checklist.

Statistics

For all statistical analyses, confirm that the following items are present in the figure legend, table legend, main text, or Methods section.

n/a	Confirmed	
☐	☒	The exact sample size (n) for each experimental group/condition, given as a discrete number and unit of measurement
☐	☒	A statement on whether measurements were taken from distinct samples or whether the same sample was measured repeatedly
☐	☒	The statistical test(s) used AND whether they are one- or two-sided<br>*Only common tests should be described solely by name; describe more complex techniques in the Methods section.*
☒	☐	A description of all covariates tested
☐	☒	A description of any assumptions or corrections, such as tests of normality and adjustment for multiple comparisons
☐	☒	A full description of the statistical parameters including central tendency (e.g. means) or other basic estimates (e.g. regression coefficient) AND variation (e.g. standard deviation) or associated estimates of uncertainty (e.g. confidence intervals)
☐	☒	For null hypothesis testing, the test statistic (e.g. F, t, r) with confidence intervals, effect sizes, degrees of freedom and P value noted<br>*Give P values as exact values whenever suitable.*
☒	☐	For Bayesian analysis, information on the choice of priors and Markov chain Monte Carlo settings
☒	☐	For hierarchical and complex designs, identification of the appropriate level for tests and full reporting of outcomes
☒	☐	Estimates of effect sizes (e.g. Cohen's d, Pearson's r), indicating how they were calculated

Our web collection on statistics for biologists contains articles on many of the points above.

Software and code

Policy information about availability of computer code

Data collection	no software was used for data collection
Data analysis	fastQC (v. 0.11.9) was used to perform quality control of RNAseq data. RNAseq fastq files were aligned to GENCODE GRCm38 and quantified with Salmon (v. 0.14.1). Differential expression analysis was performed using DESeq2 (v. 1.26.0). Gene ontological feature and signaling pathway enrichment analysis was performed by using GSEA (v. 4.0.0).<br>The CYTOF data processing and data transformation were done by flowCore , by which the raw marker intensities were Arcsinh-transformed (with cofactor 5) for cell clustering and further quantile-transformed (1% and 99% percentiles as the boundary) for heatmap visualization. The clustering and cell population identification were conducted by FlowSOM (R code is available at https://github.com/SofieVG/FlowSOM) and ConcensusClusterPlus (written in R, under GPL-2, and available through the Bioconductor project at http://www.bioconductor.org/).

For manuscripts utilizing custom algorithms or software that are central to the research but not yet described in published literature, software must be made available to editors and reviewers. We strongly encourage code deposition in a community repository (e.g. GitHub). See the Nature Portfolio guidelines for submitting code & software for further information.

Data

Policy information about availability of data

All manuscripts must include a data availability statement. This statement should provide the following information, where applicable:

- Accession codes, unique identifiers, or web links for publicly available datasets
- A description of any restrictions on data availability
- For clinical datasets or third party data, please ensure that the statement adheres to our policy

The authors declare that all data supporting the findings of this study will be uploaded to public website before publication, or are available within the paper and its

Supplementary Information files or from the corresponding author upon reasonable request.

Field-specific reporting

Please select the one below that is the best fit for your research. If you are not sure, read the appropriate sections before making your selection.

☒ Life sciences ☐ Behavioural & social sciences ☐ Ecological, evolutionary & environmental sciences

For a reference copy of the document with all sections, see nature.com/documents/nr-reporting-summary-flat.pdf

Life sciences study design

All studies must disclose on these points even when the disclosure is negative.

Sample size	For in vivo and in vitro studies, sample sizes were decided based on the similar studies reported in the previous literature. Given 3-9 repeated or mice per group, we have 80% power to detect a difference in means between two groups at a significance level of 0.05 using two-sample t-test.
Data exclusions	The data of outlier point, which was further than 1.5*interquartile Range away from the mean, was excluded.
Replication	In vivo experiment was conducted at least twice at different times. For in vitro experiment, replicates were not less than three in each group. All the replications showed the similar results.
Randomization	mice at same age and same gender with similar tumor burden were randomly grouped into different treatment.
Blinding	Mice in different groups were received different treatment performed by us. But the survival time was determined by the moribund condition which was assessed by staff works in Department of Veterinary Medicine and Surgery, who didn't know the group information of the mice (so it's single-blinded).

Reporting for specific materials, systems and methods

We require information from authors about some types of materials, experimental systems and methods used in many studies. Here, indicate whether each material, system or method listed is relevant to your study. If you are not sure if a list item applies to your research, read the appropriate section before selecting a response.

Materials & experimental systems

n/a	Involved in the study	
☐	☒	Antibodies
☐	☒	Eukaryotic cell lines
☒	☐	Palaeontology and archaeology
☐	☒	Animals and other organisms
☐	☒	Human research participants
☒	☐	Clinical data
☒	☐	Dual use research of concern

Methods

n/a	Involved in the study	
☒	☐	ChIP-seq
☐	☒	Flow cytometry
☒	☐	MRI-based neuroimaging

Antibodies

Antibodies used

CD4-PE-CY7 (clone: RM4-5) Tonbo Biosciences 60-0042-U100
CD8α-VF450 (clone: 53-6.7) Tonbo Biosciences 75-0081-U100
CD3-APC/CY7 (clone: 17A2) Tonbo Biosciences 25-0032-U100
CD8α-APC/CY7 (clone: 53-6.7) Tonbo Biosciences 25-0081-U100
CD8α-FITC (clone: 53-6.7) BioLegend 100706
CD8α-PE (clone: 53-6.7) Tonbo Biosciences 50-0081-U100
CD8β-PE (clone: YTS156.7.7) BioLegend 126607
CD11b-PE (clone: M1/70) Tonbo Biosciences 50-0112-U100
CD11c-FITC (clone: N418) BioLegend 117305
Ly6G-APC/CY7 (clone: 1A8) BioLegend 127623
CD19-FITC (clone: 1D3) Tonbo Biosciences 35-0193-U025
CD45-Pacific blue (clone: 30-F11) BioLegend 103125
TNFα-PE (clone: MP6-XT22) BioLegend 506306
Granzyme B-PE (clone: QA16A02) BioLegend 372208
IFNγ-VF450 (clone: XMG1.2) Tonbo Biosciences 75-7311-U100
CD69-FITC (clone: H1.2F3) BioLegend 104506

	CD103-PE (clone: 2E7) BioLegend 121406<br>Annexin V-Pacific blue BioLegend 640918<br>Fixable Viability Dye eFluor™ 780 ThermoFisher 65-0865-14<br>α-GAPDH (clone: D16H11) Cell Signalling Technology #5174<br>α-β-Actin (clone: 13E5) Cell Signalling Technology #4970<br>α-PIDD (clone: Anto-1) Novus Biologicals, Inc NBP1-97595
Validation	All antibodies' validation statements for the species and application can be found on the manufacturer's website.

Eukaryotic cell lines

Policy information about cell lines

Cell line source(s)	DBT mouse glioma cells were kindly provided by Dr. Leonid Metelitsa (Baylor College of Medicine). GL261 cells were obtained from the National Cancer Institute. 4T1 cells were purchased from ATCC.
Authentication	Cells were collected for DNA isolation, and further gene fingerprint authentication.
Mycoplasma contamination	All cells were treated with mycoplasma removal agent (Bio-Red) for one week and tested negative for mycoplasma contamination before use.
Commonly misidentified lines<br>(See ICLAC register)	No commonly misidentified cell lines were used in the study

Animals and other organisms

Policy information about studies involving animals; ARRIVE guidelines recommended for reporting animal research

Laboratory animals	We purchased Balb/c, C57BL/6 (5–8 weeks of age), B6.129P2-Cxcr3tm1Dgen/J, and Thy1.1 (CD90.1+) mice from The Jackson Laboratory. FcyRIIB-/- mice (Fcgr2b-Model 579) were purchased from Taconic. FGL2−/− mice were a gift from Dr. Gary Levy (Toronto General Hospital/Research Institute), and NOD.CB17-Prkdcscid/J (SCID) mice were a gift from Dr. Richard Gorlick (The University of Texas MD Anderson Cancer Center). All mice were aged 6 to 12 weeks when the experimental procedures began. Both gender were used in experiments.
Wild animals	The study didn't use wild animals.
Field-collected samples	The study did not involve samples collected from field.
Ethics oversight	We performed all animal experiments in accordance with the guidelines approved by the Institutional Animal Care and Use Committee (IACUC) at MD Anderson.

Note that full information on the approval of the study protocol must also be provided in the manuscript.

Human research participants

Policy information about studies involving human research participants

Population characteristics	The tumor tissue sections used in this study were from newly diagnosed primary GBM patients.
Recruitment	Sample collection was conducted under protocol #LAB03-0687, which was approved by the Institutional Review Board of The University of Texas MD Anderson Cancer Center, after written informed consent was obtained. Patients' tumors were graded by a neuropathologist according to the World Health Organization classification.
Ethics oversight	The Institutional Review Board of The University of Texas MD Anderson Cancer Center

Note that full information on the approval of the study protocol must also be provided in the manuscript.

Flow Cytometry

Plots

Confirm that:

- [x] The axis labels state the marker and fluorochrome used (e.g. CD4-FITC).
- [x] The axis scales are clearly visible. Include numbers along axes only for bottom left plot of group (a 'group' is an analysis of identical markers).
- [x] All plots are contour plots with outliers or pseudocolor plots.
- [x] A numerical value for number of cells or percentage (with statistics) is provided.

Methodology

Sample preparation	Mouse brain tissues and LNs were minced and enzymatically digested to obtain single-cell suspensions. BILs were isolated per

	a previous protocol3. Briefly, each single-cell suspension was centrifuged through a 30% Percoll gradient at 7800×g for 30 minutes. The leukocyte layer was collected and centrifuged on a discontinuous Ficoll-Paque Plus gradient to select and purify leukocytes. Leukocytes from mouse PB were collected as previously described4. Fc receptors were blocked using a rat anti-mouse anti-CD16/CD32 antibody (clone: 2.4G2, BD Biosciences). The antibodies used for flow cytometry are listed in Extended Data Table 1. The cell surfaces were stained by using a standard protocol. For intracellular staining, cells were fixed, permeabilized, and incubated with antibodies against IFNγ, TNFα, and granzyme B. For apoptosis assay, 7-AAD (Tonbo Biosciences, #13-6993-T500), and Annexin V antibody was used to stain cells in Annexin V binding buffer (BioLegend, #422201) for 15 min before detecting. Stained cells were isolated by flow cytometry and the results analyzed by .
Instrument	BD LSRFortessa™ - BD
Software	FlowJo software (version 10)
Cell population abundance	More than 95% cells are positive for the sorted markers, as verified by running flow after sorting to confirm the sorting efficiency.
Gating strategy	FSC-low and SSC-Low events were excluded as they were known cell debris; SSC-Hi and FSC-Low events were excluded as they were dead cells; only SSC-medium, FSC-medium events were gated for later analysis for different immune populations. Negative gating was gated by non-stained samples, or isotype antibodies stained samples; and positive gating was based on negative gating.

☒ Tick this box to confirm that a figure exemplifying the gating strategy is provided in the Supplementary Information.
